# PS^2^MS: A Deep Learning-Based Prediction System for Identifying New Psychoactive Substances Using Mass Spectrometry

**DOI:** 10.1021/acs.analchem.3c05019

**Published:** 2024-03-15

**Authors:** Yi-Ching Lin, Wei-Chen Chien, Yu-Xuan Wang, Ying-Hau Wang, Feng-Shuo Yang, Li-Ping Tseng, Jui-Hung Hung

**Affiliations:** † Department of Laboratory Medicine, Kaohsiung Medical University Hospital, Kaohsiung Medical University, Kaohsiung 807, Taiwan; ‡ Department of Laboratory Medicine, School of Medicine, College of Medicine, Kaohsiung Medical University, Kaohsiung 807, Taiwan; § Doctoral Degree Program of Toxicology, College of Pharmacy, Kaohsiung Medical University, Kaohsiung 807, Taiwan; ∥ Department of Medical Research, Kaohsiung Medical University Hospital, Kaohsiung Medical University, Kaohsiung 807, Taiwan; ⊥ Department of Computer Science, National Yang Ming Chiao Tung University, HsinChu 300, Taiwan; # Department of Medicinal and Applied Chemistry, Kaohsiung Medical University, Kaohsiung 807, Taiwan; ∇ Program in Biomedical Artificial Intelligence, National Tsing Hua University, HsinChu 300, Taiwan

## Abstract

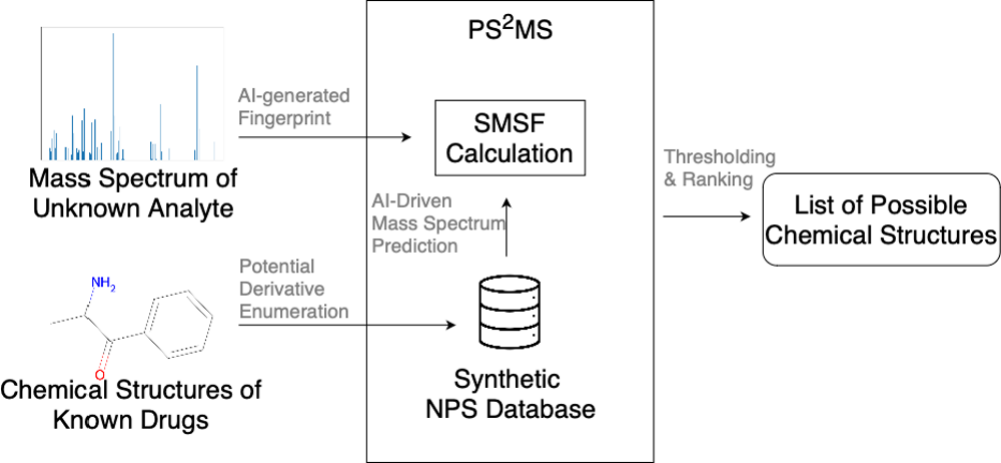

The rapid proliferation of new psychoactive substances (NPS) poses significant challenges to conventional mass-spectrometry-based identification methods due to the absence of reference spectra for these emerging substances. This paper introduces PS^2^MS, an AI-powered predictive system designed specifically to address the limitations of identifying the emergence of unidentified novel illicit drugs. PS^2^MS builds a synthetic NPS database by enumerating feasible derivatives of known substances and uses deep learning to generate mass spectra and chemical fingerprints. When the mass spectrum of an analyte does not match any known reference, PS^2^MS simultaneously examines the chemical fingerprint and mass spectrum against the putative NPS database using integrated metrics to deduce possible identities. Experimental results affirm the effectiveness of PS^2^MS in identifying cathinone derivatives within real evidence specimens, signifying its potential for practical use in identifying emerging drugs of abuse for researchers and forensic experts.

## 1 Introduction

The relentless emergence of illicit drugs, often in the form of new psychoactive substances (NPS), presents a critical threat to health and security in recent decades.^1,2^ Conventionally, illicit drugs are identified using mass spectrometry (MS)-based methods. Various techniques, such as high-performance liquid chromatography (HPLC), gas chromatography (GC), mass spectrometry (MS), ion-mobility spectrometry, Raman spectroscopy, and nuclear magnetic resonance (NMR) spectroscopy, are used to accurately detect illegal drugs present in the system.^3,4^

Among these techniques, gas chromatography/electron impact (GC/EI)-MS is one of the most widely used techniques for identifying illicit drugs.^5^ The consistent electron energy of 70 eV in GC/EI-MS is beneficial for ensuring reproducible fragmentation, which is essential for the accurate identification of NPS under varied experimental setups and across different instrument brands. The spectra are initially compared with reference spectrum libraries such as the National Institute of Standards and Technology (NIST),^6^ Wiley,^7^ and SWGDRUG^8^ MS libraries, which contain known substances. For further confirmation, these preliminary identifications are then validated against corresponding reference standards with a detailed analysis of both retention time and mass spectra to ensure precise identification.

NPS are a heterogeneous group of substances often known as either designer or synthetic drugs or “legal highs”. Traditional methods for illicit drug identification face significant challenges due to the emergence of NPS, which is designed by the structural alternations of original psychoactive substances to maintain the psychoactive effects while producing distinct mass spectra, thereby evading detection through conventional MS-based methods. For example, synthetic cathinones, a prominent class of NPS, bear a structural resemblance to substituted phenylethylamine and elicit comparable stimulating effects such as euphoria, heightened sensory perception, enhanced sociability, increased energy, mental stimulation, empathic connection, and heightened libido, similar to amphetamine.^9−12^ Notably, synthetic cathinones have emerged as the most commonly seized class of NPS, as reported by the United Nations Office on Drugs and Crime (UNODC) Early Warning Advisory System.^13^

One strategy for uncovering the identities of NPS that have managed to elude detection involves predicting and synthesizing potential analogues. Adams et al. reported a “zombie” outbreak event in July 2016 due to the newly synthetic cannabinoid AMB-FUBINACA (methyl 2-(1-(4-fluorobenzyl)-1H-indazole-3-carboxamido)-3-methylbutanoate) intoxication. The prediction and synthesis of possible cannabinoid analogues as analytical standards before their appearance in the market shorten the time of new designer drug identification.^14^ While this approach effectively addresses a cluster of mass intoxication cases, it has inherent limitations. The requirement for the expertise of a synthetic organic chemist and the generation of numerous potential derivatives for each illicit drug make the comprehensive characterization of these substances in clinical laboratories highly impractical.^15^

In fact, recent advancements in machine learning have enabled the in silico prediction of EI mass spectra. For example, neural electron–ionization mass spectrometry (NEIMS)^16^ utilizes neural networks to convert chemical structure representations (i.e., extended circular fingerprints; ECFPs^17^) into mass spectra, enabling researchers to expand the libraries with compounds with known structures. Directed message passing neural networks (D-MPNN^18^) operate on molecular graphs based on chemical structures in the simplified molecular input line entry system (SMILES^19^) notation to build neural representations of molecules for property prediction including mass spectra. Notably, a recent study by Yang et al.^20^ addresses limitations in reference library coverage by predicting additional missing mass spectra with known structures using NEIMS.

However, it is important to note that these advancements are limited to cases where the chemical structure of NPS is already known. Efforts to directly predict the structure of emerging NPS are still in its early stages. DarkNPS,^21^ for example, is a generative neural network model capable of forecasting potential new drugs by using structural formulas from reference drugs as input. Nonetheless, this approach is guided by the structural prior trained on known compounds, making it less effective in predicting emerging drugs undergoing deliberated modifications.

**Figure 1:**
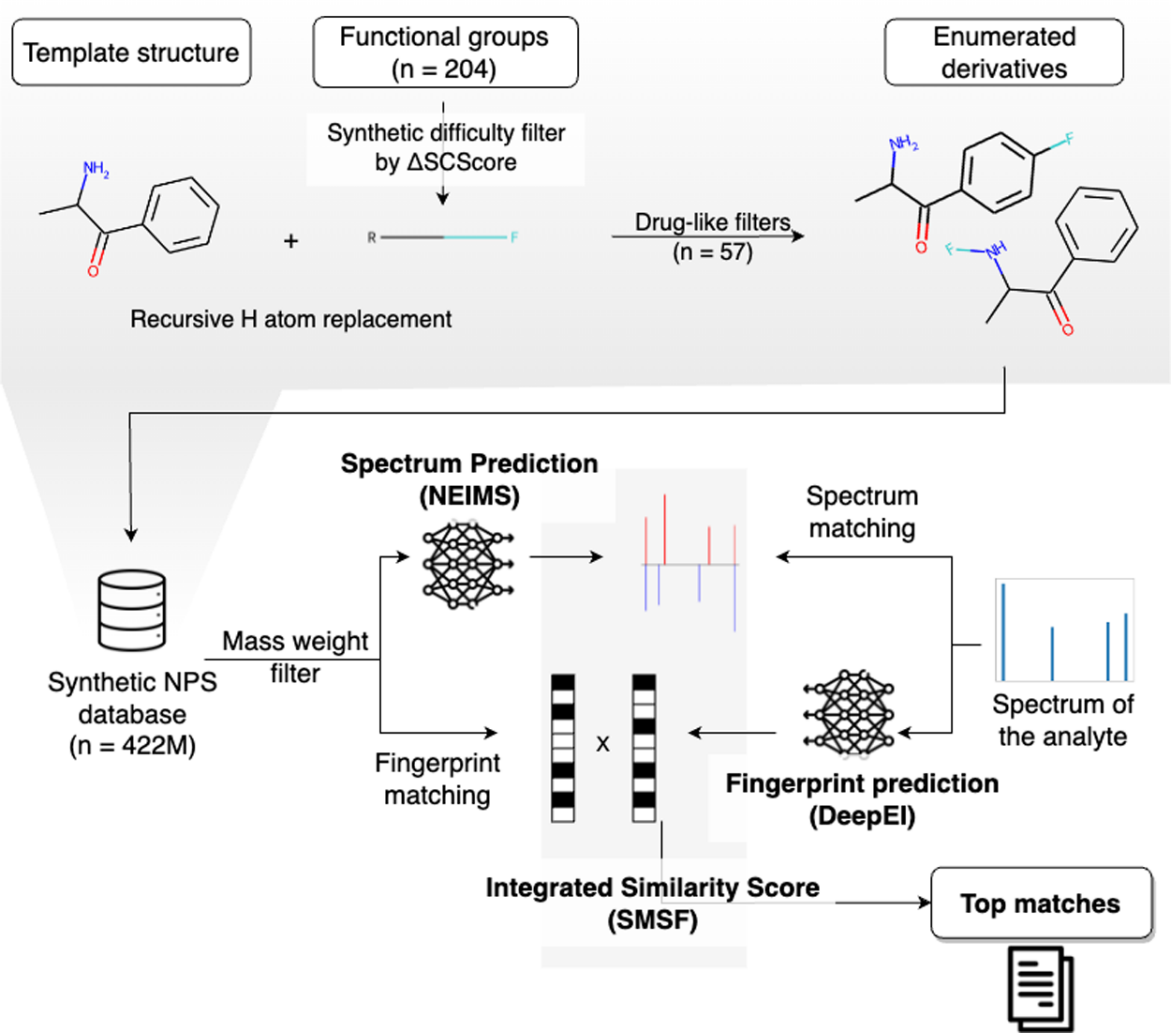
Systems overview. PS^2^MS employs an AI-driven process for unknown substances that do not match reference spectra. It generates potential derivatives from a template, filters out challenging functional groups using SCScore, screens for stability and pharmaceutical relevance, predicts mass spectra and fingerprints using two deep-learning models, calculates SMSF, and ranks potential substances. The system then determines if the analyte is a derivative of the target drug.

To tackle the challenges in identifying potential novel illicit drugs, we introduce PS^2^MS, an AI-powered predictive system, designed specifically for this purpose. When the mass spectrum of an analyte, retrieved via GC/EI-MS, does not match any established reference spectra during the conventional screening procedure, PS^2^MS builds a supplementary synthetic NPS database by enumerating possible derivatives based on the core structure of a preselected illicit drug. The system leverages two deep learning models, NEIMS and DeepEI, to predict the mass spectrum of the enumerated substances in the NPS database and the structure representations (i.e., fingerprints) of the unidentified analyte, respectively. The integrated similarity metric between the analyte and enumerated compounds in the NPS database based on the mass spectrum and fingerprint are calculated, summarized, and ranked. The system subsequently yields a list of potential NPS identities for the analyte (Figure 1). Analytic laboratories can use the provided chemical structure formulas with the highest rankings for further synthesis and research.

The proposed system was validated with spectra obtained from real-world samples, showcasing its capability to accurately analyze and identify substances. By integrating advanced computational models with practical testing, our system paves the way for innovative solutions in spectral analysis, enhancing the identification process in complex, real-world scenarios.

## 2 Methods

### 2.1 Rationale

NPS are typically derived through the structural modification of existing illegal substances as a means to circumvent law enforcement.^22^ This process involves altering molecular structures, functional groups, or substituents to create new derivatives that may exhibit similar or even enhanced potency. For instance, known amphetamine derivatives such as MDMA (3,4-methylenedioxymethamphetamine), MDEA (3,4-methylenedioxyethylamphetamine), and 6-MAPDB (1-(2,3-dihydrobenzofuran-6-yl)-*N*-methylpropan-2-amine), which belong to the amphetamine-type stimulant (ATS) class, can be produced by making basic modifications to the original amphetamine structure (Figure S1).

Leveraging the power of deep learning, we now possess the capacity to extrapolate a multitude of physical and chemical properties from simplified structural representations. This technological advancement enables us to expand conventional reference spectra with AI-generated supplements, representing feasible derivatives of known illicit substances. PS^2^MS specifically fulfills two critical yet missing components for the purpose:

1. Comprehensive Enumeration: It systematically catalogues potential NPS compounds resulting from structural modifications of established illegal substances.
2. Effective Matching Metric: It provides a practical and efficient method to suggest and rank potential identifications for the given analyte.

The augmentation substantially advances the detection of emerging NPS by integrating traditional mass spectroscopy with our predictive system, as depicted in Figure 1. Validation procedures can then be carried out with guidance from the recommendations provided by our predictive system.

### 2.2 Enumeration and Rule-Based Filtering of Derivatives

To begin with, the chemical structure of a preselected known drug was used as a template for enumerating candidate derivatives. The core of the initial structure, encompassing all heavy atoms and their bonds within the molecule, was maintained, and only the hydrogen atoms within the molecule underwent iterative chemical modification. A total of 204 common functional groups were compiled unbiasedly (see Table S1 and Section 4), and the R-group of each functional group was concatenated with the atom connected to the hydrogen slated for replacement, serving as a chemical modification. This hydrogen-atom-functional group replacement process was performed recursively on a structure to include derivatives with multiple chemical modifications.

**Figure 2:**
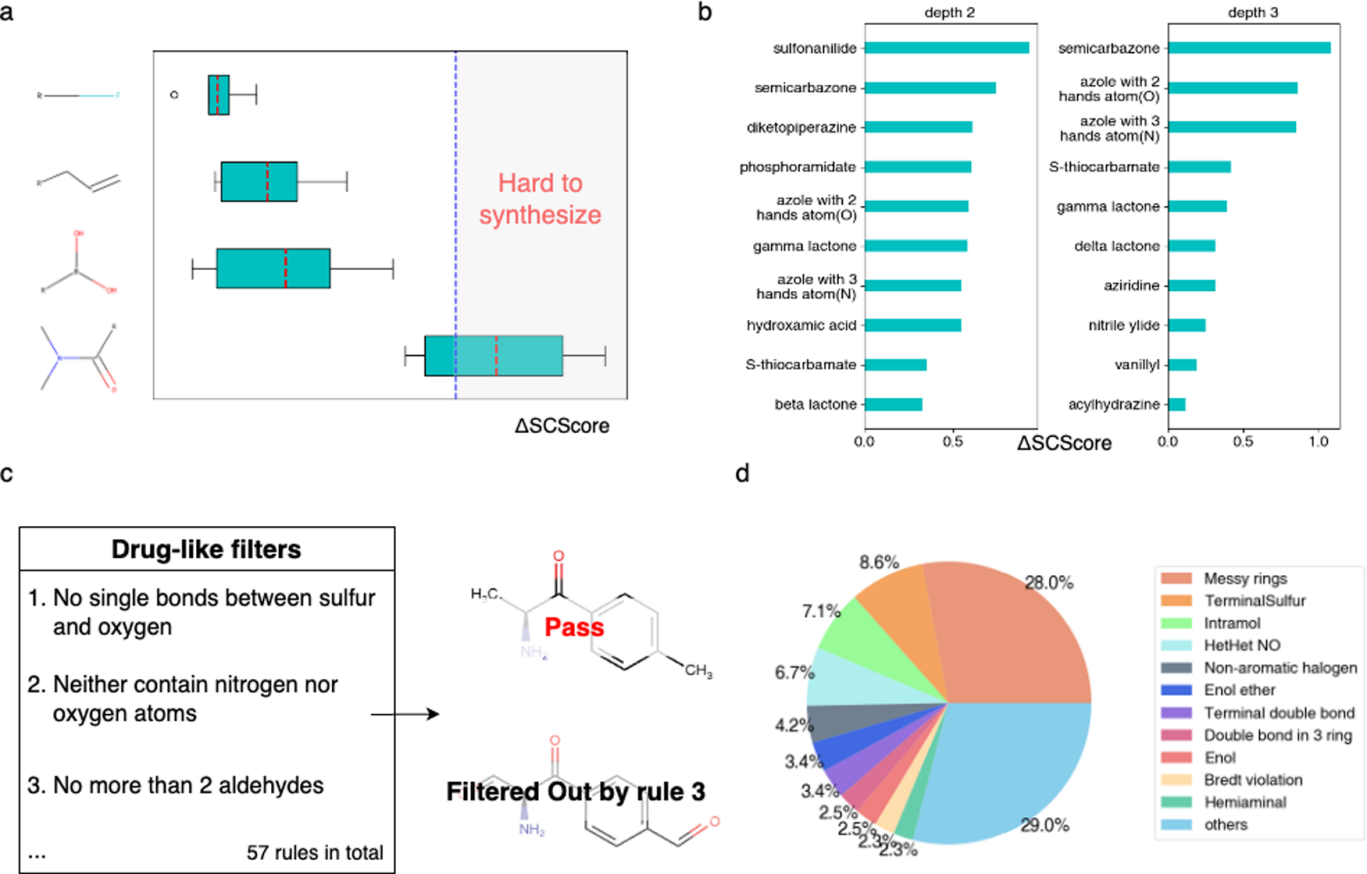
Derivative filtering. (a) SCScore is employed to evaluate the average synthesis difficulty of the target drug after the addition of functional groups. The *x*-axis portrays the score difference achieved by subtracting the SCScore of the target drug (i.e., ΔSCScore), while the *y*-axis presents the functional group. (b) At each level, a filtering process is applied to the number of functional groups. The 10 filtered functional groups with the highest ΔSCScore in the 2nd and 3rd recursion are listed. (c) After enumerating compounds, a set of 57 rules are applied to filter out unstable or nonpharmaceutical enumerations, ensuring that only drug-like compounds are retained. (d) Proportion of compounds filtered out by each rule among the set of 57 rules.

The depth of recursion increases the comprehensiveness of the derivatives and also leads to exponential growth in the enumeration space. Since some of the 204 chemical modifications are considerably more challenging to synthesize than others, and derivatives involving many of these complex modifications are unlikely to be practically synthesized, such modifications can be excluded during recursion to prevent excessive enumeration. To identify these challenging chemical modifications, at each functional group within the nth (*n* > 1) recursive depth, we iteratively modified the target compound with that functional group *n* times. We then calculated the average synthetic complexity score of the resulting compounds using SCScore.^23^ SCScore is a neural-network-based model that provides a score based on the expected number of reaction steps required to synthesize a compound. Subsequently, we utilized the synthesis difficulty score of the template drug as the baseline and retained only the functional groups whose average synthesis difficulty differential (represented as ΔSCScore) fell below a threshold of α^*n*^ (as shown in Figure 2a).

In our experiment involving the enumeration of cathinone derivatives, we set α = 1.2 and *n* = 3, which allowed us to enumerate all known illicit cathinone-type drugs listed in the United Nations Office on Drugs and Crime (UNODC) database^24^ (Figure S2). The number of functional groups that pass the threshold in the second and third recursion levels is 131 and 97, respectively. A summary of the functional groups that were filtered according to their ΔSCScore can be found in Figure 2b. It is worth noting that α should be set greater than 1 and the higher parameter values yield more comprehensive derivative candidates but may also require substantial processing time in subsequent steps (see below).

We further introduced 57 drug-like criteria according to Virshup et al.^25^ (see Supplementary Methods) that take more fundamental properties in practical aspects into account, such as the stability of the structure or the suitableness of being a drug, to eliminate derivatives after the enumeration (Figure 2c). After conducting the screening process, we noticed that some rules filtered out significantly more molecules than others. The most effective rule was Messy rings (more than 7 smallest sets of smallest rings) as shown in Figure 2d. By prioritizing the most effective rules, we can optimize the screening process and save computational resources, ultimately improving the screening efficiency.

The remaining derivatives were compiled from the NPS database. In our experiments with cathinone, the process of enumeration and filtering resulted in a total of approximately 422 million compounds.

### 2.3 Mass Spectrum Generation of Derivatives in the NPS Database

To facilitate a direct comparison with the mass spectrum of the unknown analyte by using EI-MS, we employed NEIMS to predict the mass spectrum of all derivatives within our synthetic NPS database.

**Figure 3:**
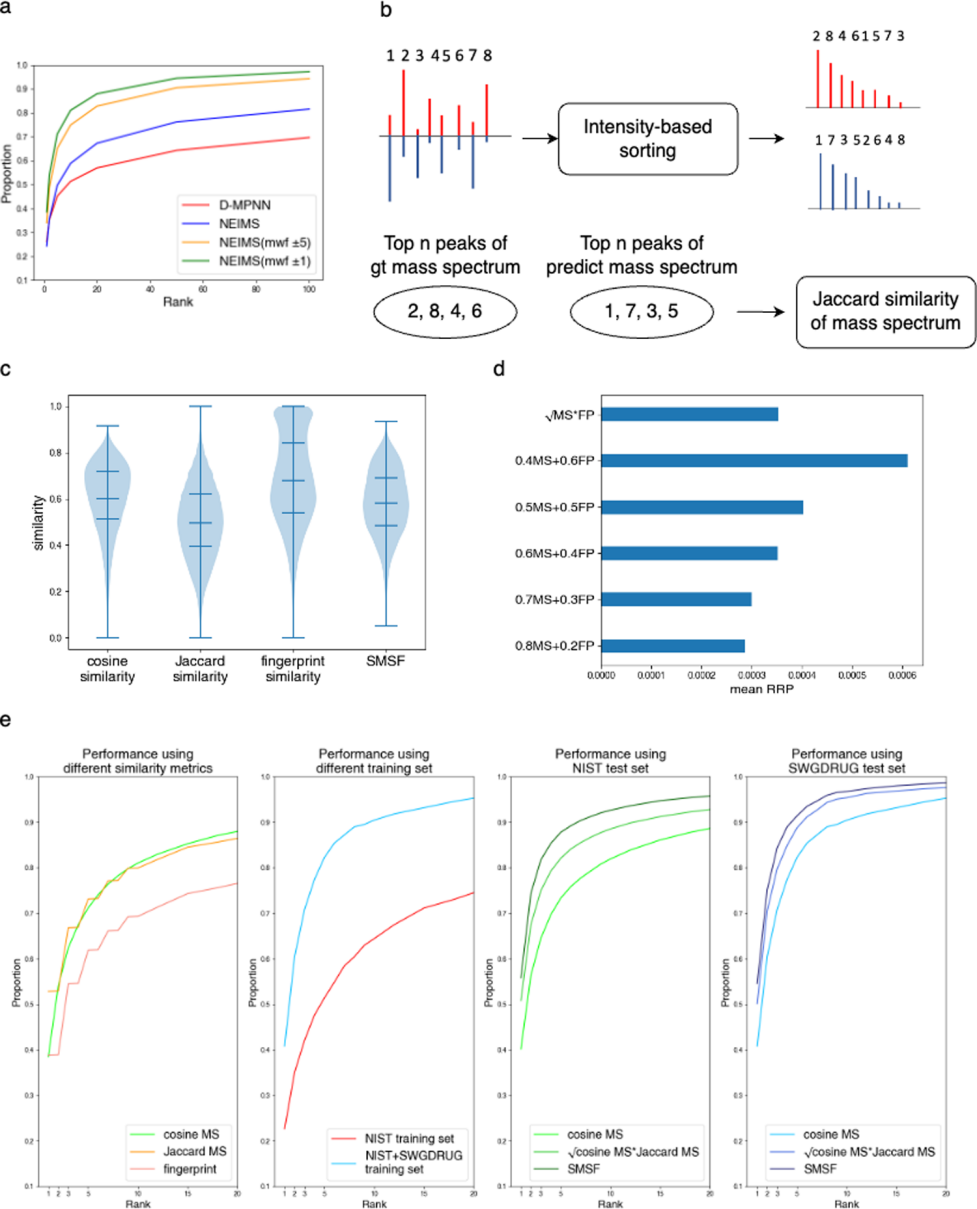
(a) Ranking proportion curves of the test substance among the models are depicted. D-MPNN is the baseline model for reference. mwf stands for the mass weight filter. (b) Method for calculating the top *n* Jaccard similarity of two mass spectra, where *n* is 4. (c) Distribution of different similarity metrics between the true and predicted data for the NIST test set. (d) Evaluation of the NIST test set ranking performance using various calculation methods. The *x*-axis represents the mean RRP, which measures the quality of the ranking. A value closer to 0 indicates better ranking performance, while an RRP value of 0.5 suggests random performance. (e) Comparison of ranking performances.

As the original NEIMS was initially trained solely using spectra from the NIST library, which covered a broader range of chemicals yet with only a small fraction dedicated to drugs, its performance significantly declined when tested with spectra of illicit drugs in our experiments (Figure 3e). To address this, we obtained mass spectrum data from the Scientific Working Group for the Analysis of Seized Drugs (SWGDRUG) for training and testing NEIMS. We then meticulously sampled, partitioned, and subsequently retrained NEIMS. The retrained NEIMS demonstrated comparable performance when tested on a mixture of both NIST and SWGDRUG data sets using the same metrics (with different mass weight filters, i.e., mwf ± 1 and 5) as reported in the original paper^16^ (see Figure 3a).

In practice, the molecular weight is typically inferred from the M+ peak in a mass spectrum; however, in EI-MS, this peak may not be present due to the technique’s hard ionization nature. To circumvent this, complementary techniques such as chemical ionization (CI) are recommended to obtain reliable molecular weight information (see Section 4 for more details). Additionally, the implementation of an ±1 Da tolerance in PS^2^MS is to account for isotopic variance and other artifacts specific to accurate spectrum prediction by NEIMS, rather than to compensate for missing M+ peaks. This tolerance is applied throughout our system evaluations to enhance the practicability and performance.

### 2.4 Enhancing Similarity Metrics for Spectrum Matching

Given the vast number of derivatives in the synthetic NPS database—totaling hundreds of millions—precise spectrum matching necessitates a highly specific metric. Although cosine similarity is a common choice for comparing two spectra, its design overlooks certain prominent features often present in the spectrum of a particular compound.^26^ Notably, manually analyzing spectra often begins with identifying the highest intensity peaks at specific *m*/*z* ratios; however, this feature is not extensively factored into the calculation of cosine similarity.

To address this, PS^2^MS adopts Jaccard similarity to assess the overlap between the top peaks of two spectra, enhancing the system’s capacity to better capture crucial characteristics. The distribution of metrics used to match between the predicted and true spectra is depicted in Figure 3c. In our experiments, the integration of cosine similarity and Jaccard similarity led to improved accuracy in predicting mass spectra especially in the top ranks (see Figure 3e), when testing with the selected substances in the NIST and SWGDRUG data sets. The proportion of correct prediction appearing at top 1 increased from below 40% to more than 50% after incorporating Jaccard similarity.

### 2.5 Integrated Similarity Metric Based on Mass Spectrum and Molecular Fingerprint

In addition to the direct matching of mass spectra, PS^2^MS employs molecular fingerprints^27,28^ to further facilitate the screening of the analyte’s potential identity. A compound’s fingerprint is a binary sequence composed of 0s and 1s, containing structural information that ensures distinct fingerprints for different compounds. Each compound possesses a unique fingerprint, which can be derived by converting its structural representation.^29^ The fingerprints of all of the derivatives in the NPS database can be easily transformed using their known structure representation during enumeration.

However, since the structure of the analyte is absent, PS^2^MS employs the DeepEI^30^ deep learning model to convert the analyte’s mass spectrum into its corresponding fingerprint, all without the need for explicit structure information. A similarity metric based on the fingerprints of both the analyte and its derivatives can then be readily calculated.

To validate the effectiveness of this strategy, we evaluated the performance of fingerprint-based identification using the NIST and SWGDRUG data sets (see Figure 3e). While fingerprint-based identification alone did not exhibit superior effectiveness compared to spectrum-based identification, Ji et al.^30^ suggested that the accuracy can be enhanced by combining the similarity metrics of fingerprints and mass spectrum. Therefore, we explored various combinations of these metrics and evaluated them using the relative ranking performance (RRP) outlined in ref (31). We denote the best-performing integrated similarity metric (i.e., a weighted **s**um of the **m**ass **s**pectrum and **f**ingerprint similarity scores) as SMSF (see Supplementary Methods, Figure 3c,d).

Utilizing SMSF as the metric to guide the prioritization of matching derivatives led to further enhancement in performance. In the NIST and SWGDRUG data sets, over 90% and 85% of the test substances, respectively, ranked within the top 20 predictions (Figure 3e). The results demonstrate the feasibility of using a predicted mass spectrum and fingerprint for precise compound identification within the synthetic NPS database.

## 3 Results

### 3.1 Anonymized Testing of Known Cathinone Derivatives

To evaluate the efficacy of PS^2^MS, we anonymized 20 known derivatives of cathinone (i.e., positive samples) and 20 noncathinone (i.e., negative samples) that belong to derivatives of amphetamine (*n* = 7), phencyclidine (a.k.a. PCP, *n* = 1), psychedelics (a.k.a. 2C, *n* = 2), and cannabis (*n*– = 10) and generated their mass spectra using standard references from EI-MS. Since amphetamine derivatives are closely related to and structurally similar to cathinone, they can be considered challenging to the predictive system. The 40 mass spectra were then compared to the synthetic NPS database utilizing cathinone as the enumeration template. Note that the spectra of substances in these 40 known samples were removed from the training of the NEIMS to make sure they are invisible to PS^2^MS.

As PS^2^MS prioritizes derivatives based on the SMSF score, establishing a threshold becomes crucial to signify a “no match” when analytes do not resemble any derivatives. By forming Gaussian distributions of similarity scores for cathinone-like and noncathinone-like drugs from SWGDRUG data sets, the threshold was defined at the point where two probability functions intersect. We found that a similarity threshold of 0.55 can effectively differentiate between similar and dissimilar substances (see Figure S3). Only derivatives with an SMSF score above the threshold are deemed to be matches.

**Figure 4:**
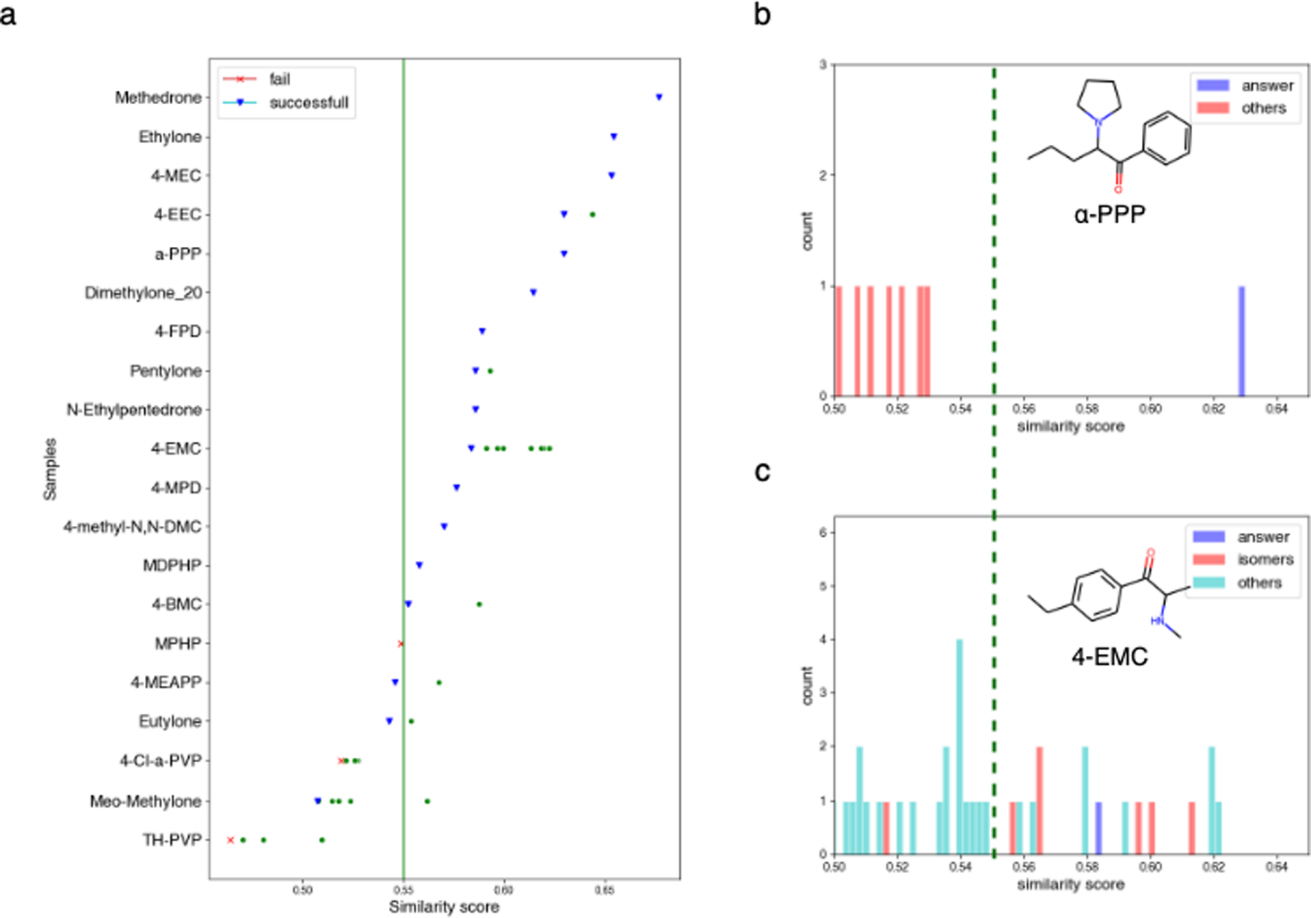
(a) Performance on 20 positive samples. The *x*-axis represents the similarity score. The blue inverted triangle indicates successful identification of the sample as an illicit drug, while the red cross signifies failure in determining the sample as such. The green circles in the figure represent the compounds in the database that exhibit higher similarity than those in the answer. (b) Among the enumerated cathinones, the similarity score distributions for the cathinone-like drug α-PPP. (c) Among the enumerated cathinones, the similarity score distributions for the cathinone-like drug 4-EMC. The isomers of 4-EMC in the enumerated database are marked.

For the 20 positive samples, PS^2^MS successfully reported 17 of them as cathinone derivatives (see Figure 4a). The majority of the correct identity appeared at top 1 (10 out of 17) and top 2 (5 out of 17), suggesting that the downstream validation of the derivatives listed in higher ranks can be very effective. Notably, a clear separation in SMSF score between the top 1 prediction and others often indicates the correct prediction (Figure 4b). Interestingly, instances with multiple leading predictions without distinct separation often represent isomers (Figure 4c). It is possible by applying additional postprocessing, these features can provide valuable information for further examination to determine precise identities.

Among the 3 false negatives (i.e., MPHP, 4-CL-α-PVP, and TH-PVP), despite their correct predictions being at high ranks (see Figure 4a), they did not surpass the similarity threshold. The highest similarities of the three false negatives are 0.548, 0.525, and 0.509, respectively. In the future, we anticipate the potential incorporation of additional features to enhance prediction accuracy (see Section 4).

On the other hand, 18 out of the 20 tests conducted on noncathinone drugs, only two test substances had a similarity score higher than the threshold, leading to misjudgment as a cathinone derivative (Figure S4a). The two false positives PS^2^MS predicted, MAPDB and MMA, are both amphetamine-like drugs and show highly similar features with cathinone (Figure S4b,c). Their highest similarities for MAPDB and MMA are 0.628 and 0.553, respectively. The top 10 hits of MAPDB are actually the isomers of MAPDB, similar to that of the false negatives.

Overall, the specificity and sensitivity of PS^2^MS for screening 20 cathinone derivatives and 20 noncathinone NPS are 0.9 and 0.85, respectively, reaching an F1 score above 0.87.

### 3.2 Case Study: Eutylone

**Figure 5:**
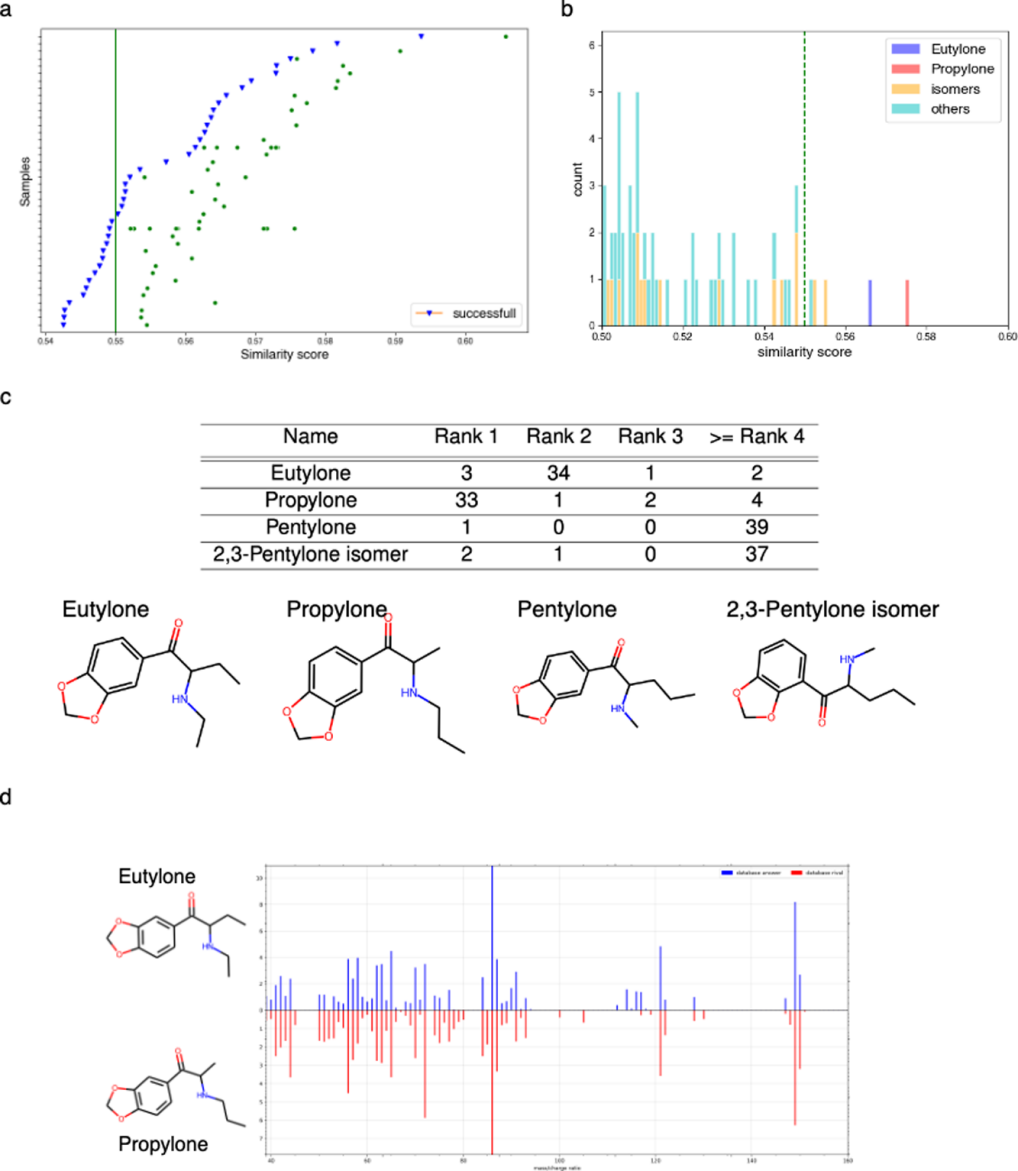
(a) Performance on 40 real samples of Eutylone. The *x*-axis represents the similarity score. The blue inverted triangle indicates successful identification of the sample as Eutylone. The green circles in the figure represent the compounds in the database that exhibit higher similarity than the corresponding answer. (b) Distribution of the most similar similarity scores for one sample of Eutylone. (c) Among the 40 samples of Eutylone, the frequently occurring top-ranked compounds are observed alongside their corresponding structures. (d) Comparison of the predicted mass spectra between Eutylone and Propylone.

Although PS^2^MS successfully detected Eutylone in the experiments above using a standard reference, its SMSF score (0.5537) is in fact at the borderline of the cutoff (0.55). We further collected 40 real-world seized samples, comprising 35 coffee pods, three tablets, one liquid, and one plastic bag, each exhibiting varying purity levels of Eutylone. These samples were subjected to typical GC/EI-MS analysis. A match was declared if the mass-to-charge (*m*/*z*) ratios of the three highest intensities aligned. The resulting spectra were then sent to PS^2^MS to ascertain whether they could be derivatives of cathinone. Impressively, all spectra were successfully identified, with 37 out of 40 samples correctly ranked within the top 3 matches (Figure 5a).

Upon closer analysis, we observed a recurring pattern where two prominent matches consistently stood out from other predictions (Figure 5b). Besides the correct identification of Eutylone, it was Propylone that frequently ranked as the top 1 prediction, even surpassing Eutylone in most of the instances (see Figure 5c). Propylone is an isomer of Eutylone, differing only in the position of the methyl group. Due to the striking similarity between the predicted mass spectra of Eutylone and Propylone (see Figure 5d), distinguishing between them in testing poses a considerable challenge for PS^2^MS at its current stage (see Section 4). Nevertheless, it is important to note that PS^2^MS still successfully identified all 40 samples as derivatives of cathinone, even if it occasionally failed to rank Eutylone as the top 1 prediction. This underscores the system’s effectiveness when applied to real-world samples.

## 4 Discussion

PS^2^MS exhibits significant potential for widespread adoption in conventional EI-MS-based NPS detection procedures. When a sample does not match known references, it can be submitted to PS^2^MS to determine whether it contains derivatives of known illicit drugs. This system can expand the synthetic NPS database by incorporating various known drug templates. While in this study we utilized cathinone as the drug template for derivative enumeration, it is clearly scalable to include more drug templates, thereby suggesting a wider range of NPS.

Furthermore, it is essential to emphasize that while our enumeration process centered around a specific target drug (e.g., cathinone), the spectral predicting model itself underwent training on a comprehensive data set representing a diverse array of chemicals. This inclusive training approach enables the model to learn and generalize spectral patterns beyond the confines of the target drug, enhancing its versatility and applicability.

Recognizing the importance of addressing potential challenges in the model’s predictions, particularly with an increasing number of target drugs, we envision future enhancements. We plan to explore the integration of an additional classifier, which would assess whether a substance possesses key structural features of the target drugs before matching with the corresponding enumeration in the pipeline. This proposed classifier could potentially enhance the model’s discriminatory capabilities and improve its overall performance.

PS^2^MS’s unbiased collection of chemical modifications, totaling 204 functional groups, combined with its flexible derivative enumeration and filtering processes, endows it with a comprehensive search space of possible derivatives. In fact, it is tempting to utilize a significantly smaller set of modifications for the generation of derivatives within the PS^2^MS system. In the UNODC list, there are 30 distinct functional groups that encompass all known cathinone-type drugs. If we were to employ only these 30 functional groups for the enumeration process, it would significantly limit the search space, specifically 2.3 million compared to the original 420 million potential derivatives, reducing the time required from 20 h to just minutes. However, this efficiency boost can lead to instances where potential compounds, which may be valid derivatives, are not included in the results due to the limited scope of functional groups considered. While it is possible to use a smaller set for generating derivatives, employing the complete set is recommended despite longer enumeration times as it reduces the risk of false negatives.

Notably, during enumeration, PS^2^MS employs the SCScore to assess the difficulty of synthesis of the target drug. This information is then used to calculate the synthesis difficulty of the enumerated compounds after the addition of functional groups. We utilize these scores to screen functional groups, thus avoiding the inclusion of highly challenging-to-synthesize compounds in the enumeration process. It is important to note that the scoring standards for synthesis difficulty may vary depending on the target drug. Consequently, the screening of functional groups should be adjusted accordingly for different target drugs. Nevertheless, this adjustment is a one-time process and has minimal overall impact.

Furthermore, adopting more stringent screening conditions can reduce the number of listed compounds and enhance the calculation speed. However, there is a trade-off as it may lead to a risk of missing potential compounds. Conversely, using looser screening conditions will yield a more comprehensive list but may prolong the prediction of mass spectra and similarity calculations. The number of enumeration levels also has a similar impact, and it is possible to adjust the parameters in PS^2^MS for improved sensitivity when necessary.

During the review process of our article, a related study, NPS-MS,^32^ was published. This study advances the field by employing transfer learning to predict tandem mass spectrometry (MS/MS) spectra of NPS based on their chemical structures. Notably, a previously undetected derivative of phencyclidine in seized substances was identified by comparing observed spectra with predictions from structural generation via DarkNPS. NPS-MS constitutes a crucial development in the automated, precise identification of NPS, harnessing the full analytical potential of MS/MS technology. In line with the goals of NPS-MS, our PS^2^MS system distinctively integrates EI-MS within the GC/MS framework for NPS identification and is further enhanced by an exhaustive enumeration and filtering process, enabling a deep exploration of possible NPS derivatives.

PS^2^MS is specifically optimized for GC/EI-MS due to two main factors: First, the GC/MS instrument is more cost-effective and widely adopted than other MS/MS instruments. Second, the consistent electron energy of EI-MS, in conjunction with the universally accessible and extensive mass spectral libraries, which are more standardized than MS/MS libraries that can differ from one vendor or laboratory to another, markedly bolsters the practicality and applicability of the predictive database we have developed. The universal standardization and practicality of EI-MS enable PS^2^MS to become a significant complementary tool alongside other MS/MS databases for practical deployment in diverse analytical settings.

PS^2^MS utilizes the M+ peak as a reference for the mass weight filtering of enumerated compounds within a certain tolerance range. However, the high-energy ionization nature of EI-MS may lead to the absence of the M+ peak. To mitigate the effects of the missing M+ peak on PS^2^MS’s performance, it is advantageous that most instruments capable of electron ionization also offer chemical ionization (CI) capabilities. This dual functionality allows forensic laboratories to employ a switchable ionization source, leveraging CI’s softer ionization properties to confirm molecular masses when they are not discernible in EI-MS spectra. Such versatility in ionization techniques ensures that molecular weights can be accurately determined, thereby supporting the robust performance of PS^2^MS. In addition, recent development of computational methods offers new ways to deduce nominal molecular masses from EI-MS data, as demonstrated by Moorthy et al.,^33^ thereby enhancing the robustness of PS^2^MS amid challenges posed by ionization variations.

While EI-MS instruments often have the capacity for CI, allowing for flexibility in ionization techniques, electrospray ionization (ESI-MS) also serves as a viable alternative,^34^ particularly for preserving the M+ peak. However, while ESI-MS could theoretically be used to train predictive models, it faces practical challenges. Variations in voltage settings across different ESI-MS instruments and experiments introduce inconsistencies that pose a significant barrier to creating a uniform spectral database, which is essential for the accuracy and reliability of the predictive models.

Regarding the molecular weight filter, in practical scenarios, the analyte’s mass spectrum may be contaminated with impurities or affected by other technical confounding factors, resulting in inaccuracies in the M+ peak representation of the correct mass weight. Consequently, this can significantly impact the subsequent mass weight filtering and spectrum matching processes. When it is determined that the mass weight filter is not applicable in such cases, the filter can be lifted. However, it should be noted that PS^2^MS exhibits reduced accuracy when the mass weight filter is not employed.

In consideration of factors such as speed, accuracy, and practical feasibility for large-scale application, we determined NEIMS as the most suitable choice for our research requirements. Our choice was influenced by NEIMS’s proven efficiency and the pragmatic aspects of its integration during the development of our system. We recognize the dynamic nature of the field with ongoing advancements in EI mass spectral prediction. Emerging models like RASSP,^35^ Molformer,^36^ and BioNeMo (https://github.com/NVIDIA/NeMo/) have demonstrated promising potential, and we remain open to integrating the latest and most effective methods into our framework. As demonstrated in our experiments, the current PS^2^MS has limitations in effectively distinguishing isomers due to the lack of distinctiveness in the predicted spectra. To address this, incorporating more accurate in silico spectrum prediction models could significantly enhance PS^2^MS performance in the future.

## 5 Conclusions

In this study, we introduced a pioneering predictive system named PS^2^MS to address the challenges posed by the rapid emergence of NPS and their identification through mass spectrometry by constructing a synthetic NPS database through the enumeration of derivatives based on known substances and leveraged deep learning models such as NEIMS and DeepEI in predicting mass spectra of NPS derivatives and fingerprints of the analyte, respectively.

Our approach significantly enhanced the effectiveness of spectrum matching through the incorporation of Jaccard similarity, leading to improved accuracy in predicting mass spectra, particularly in the top ranks. The introduction of molecular fingerprints provided an additional layer of identification potential, and the development of the integrated similarity metric SMSF demonstrated its superiority in compound identification within the synthetic NPS database.

The validation of PS^2^MS was carried out through anonymized testing of known synthetic cathinones, showcasing high specificity and sensitivity. Furthermore, the case study involving eutylone highlighted the challenges associated with closely related isomers, shedding light on potential areas for future enhancement.

Overall, PS^2^MS offers the first practical approach in tackling the complex problem of new illicit drug identification using GC/EI-MS. With the rapid emergence of NPS, the conventional analytical method used to identify unknown substances has become inadequate to keep up with the pace of the development of new compounds. By harnessing the capabilities of AI and deep learning, this system holds significant promise as a potential tool in aiding researchers and forensic experts in identifying novel emerging drugs of abuse earlier and efficiently.

## Data Availability

The complete source code and script of PS^2^MS are freely accessible via the GitHub repository at https://github.com/jhhung/PS2MS. Researchers are encouraged to utilize and contribute to this open-source resource for further exploration and development.

## Supporting Information Available

Additional details of methods and materials, as well as additional figures supporting the construction and validation of the models and tables listing the chemical modifications and drug-like filter rules (PDF)
