# Supplementary material for: PS2MS: A Deep Learning-Based Prediction System for Identifying New Psychoactive Substances Using Mass Spectrometry
Source: Anal Chem. 2024 Mar 15;96(12):4835–44. doi: 10.1021/acs.analchem.3c05019 (PMC10974679; doi:10.1021/acs.analchem.3c05019)
Supplement: Supplementary file 1 — ac3c05019_si_001.pdf [file ac3c05019_si_001.pdf]

Supporting Information

PS²MS: A Deep Learning-Based Prediction System for Identifying New Psychoactive Substances Using Mass Spectrometry

Yi-Ching Lin[1,2,3,4](winterjeanne@gmail.com ORCID: 0000-0002-0849-6149),
Wei-Chen Chien[5](brian1000823@gmail.com),
Yu-Xuan Wang[5](inferno1262380@gmail.com),
Ying-Hau Wang[5](blenderwang.cs10@nycu.edu.tw),
Feng-Shuo Yang[1,6](1010001@mail.kmuh.org.tw ORCID: 0000-0001-7256-2465),
Li-Ping Tseng[1](tseng1009@gmail.com ORCID: 0000-0003-3052-4908),
and Jui–Hung Hung[5, 7]*(JuiHungHung@gmail.com ORCID: 0000-0003-2208-9213)

[1] Department of Laboratory Medicine, Kaohsiung Medical University Hospital, Kaohsiung Medical University, Kaohsiung 807, Taiwan;

[2] Department of Laboratory Medicine, School of Medicine, College of Medicine, Kaohsiung Medical University, Kaohsiung 807, Taiwan;

[3] Doctoral Degree Program of Toxicology, College of Pharmacy, Kaohsiung Medical University, Kaohsiung 807, Taiwan;

[4] Department of Medical Research, Kaohsiung Medical University Hospital, Kaohsiung Medical University, Kaohsiung 807, Taiwan.

[5] Department of Computer Science, National Yang Ming Chiao Tung University, HsinChu 300, Taiwan;

[6] Department of Medicinal and Applied Chemistry, Kaohsiung Medical University, Kaohsiung 807, Taiwan;

[7] Program in Biomedical Artificial Intelligence, National Tsing Hua University, HsinChu 300, Taiwan;

*Correspondence to: **Jui–Hung Hung.** Email: jhh@cs.nycu.edu.tw or juihunghung@gmail.com

Table of Contents

Supplementary Methods 3

Supplementary Figures..10

Supplementary Tables 13

Reference 38

Supplementary Methods

Test environment and software resource

Codes of NEIMS (https://github.com/brain-research/deep-molecular-massspec) and DeepEI (https://github.com/hcji/DeepEI) are available on their own GitHub websites. Both of the models were trained using the Taiwania2 computing nodes with 8 Nvidia Tesla V100 hosted in National center for high-performance computing (NCPC). We test the rest of our system in the x86_64 Ubuntu 20.04 LTS environment with 80 cores and 252 gigabytes of memory. The NEIMS model was trained according to the official tutorial (https://github.com/brain-research/deep-molecular-massspec/blob/main/Model_Retrain_Quickstart.md), with the training epoch sets to be 20,000 and using the mean square error as loss function. The trained model of D-MPNN is taken from the Chemprop package[4]. The code of SCScore and pre-trained models can be obtained from the GitHub website of SCScore(https://github.com/connorcoley/scscore). The fingerprints of derivatives were generated using the "get_cdk_fingerprints" function in the DeepEI suite.

Drug enumeration

To predict all potential emerging drugs of abuse, the aim is to employ the known fundamental structures of various drugs of abuse as input and perform chemical modifications without altering their primary structure. This approach is designed to prevent any changes in drug properties that could result in the loss of psychoactive effects. The molecular structure is represented using the SMILES format. To obtain the structural formula, we rely on RDKit[5], an Open-Source Cheminformatics C++ Library that provides us with the necessary data structures, such as the molecular fingerprint and mass weight. The mass weight is used by the mass weight filter (mwf) in the ranking step to only consider derivatives that weigh within a given tolerance range (± 1) of the M+ peak of the spectrum of the analyte.

This study uses a systematic approach to enumerate derivatives based on the input drug structure by removing adjacent terminal hydrogen atoms and attaching functional groups at that site to achieve chemical modification of the drug. For the purpose of achieving a comprehensive listing of all potential emerging drugs of abuse, a collection of 204 known functional group structures was gathered. However, it was found that a single modification of the target drug was

not sufficient to enumerate all possibilities for all drugs. Analyzing data from UNODC, which comprises over 200 cathinone-type listed drugs, reveals that most of these drugs necessitate three to four chemical modifications and encompass more than 30 functional groups. We found some of these listed drugs demand four rounds of functional group modifications, which include slight alterations to the carbon chain, such as adding ethyl groups. These extra functional groups can be readily synthesized and incorporated. Due to their simplicity in synthesis, they become attractive choices for drug synthesis steps. We tackled these modifications by adding additional functional groups – dimethyl, dibutyl, and dipropyl – were introduced to the functional group collection in the 3rd recursion. These supplementary functional groups, requiring two modifications each, were incorporated to prevent the omission of controlled derivative drugs. A recursive process was carried out three times to generate drug derivatives.

The 57 drug-like criteria[1] (see **Table S2**) are used to further eliminate derivatives. Critically for GC/MS, our drug-like filters address concerns related to thermal stability, volatility, and chemical inertness by implementing specific rules such as the exclusion of compounds prone to decomposition at high temperatures (Rule 7, C=N filter), avoidance of decarboxylation-prone substances (Rule 8, decarboxy filter), and filtering out large polycyclic compounds that might decompose (Rule 21, polycyclic filter). Additionally, rules targeting non-volatility, including restrictions on the number and size of rings (Rule 29 and Rule 30) and the number of rotatable bonds (Rule 50), contribute to refining compound selection for applications such as GC-MS. The consideration of chemical inertness is addressed through rules targeting potentially reactive functional groups (Rule 51) and bonds that might be reactive under GC conditions (Rule 37 and Rule 38). These drug-like filters collectively enhance the suitability and reliability of compounds in our synthetic NPS database for diverse analytical methodologies, including GC-MS.

Use SCScore to filter functional groups

To prevent the generation of derivatives that are impractical to synthesize, the SCScore model is utilized to screen functional groups. The "full_reaxys_model_1024bool" pre-trained model (downloaded from

https://github.com/connorcoley/scscore/tree/master/models) is used, with the SMILES of the compound serving as input to obtain the synthesis difficulty score of the compound.

To screen functional groups, n recursions of modification are initially performed for a functional group, potentially resulting in several derivatives due to various modification positions at each layer. For the modified derivatives of the nth recursion, individual synthesis difficulty is calculated using SCScore, and the average score is determined. Based on the synthesis difficulty of the target compound, if the average synthesis difficulty score is below $SCScores(target\ drug) + \alpha^n$, where α is a user-adjustable hyperparameter, the functional group will be added to the functional group set of the nth recursion. In this paper, α is set to 1.2. The functional group set of each recursion will be the cascade of the functional group sets below the layer. Note that all 204 functional groups are included in the first recursion regardless of their synthesis difficulty.

Candidate Selection

The ACSESS[1] algorithm's built-in screening rules are utilized, encompassing 40 rules for identifying unstable molecules and 17 rules for selecting drug molecules (see **Table S2**). This helps to reduce unnecessary computation and identify potential emerging drugs that can be prepared. The molecular structure screening method of ACSESS has been implemented using the RDKit library. RDKit was selected due to its cost-effectiveness for future use and promotion, as well as its modularization capabilities and ease of program acceleration compared to Python.

Training and Testing Datasets

In order to train the prediction model of mass spectrometry, the 2017 NIST Mass Spectral Library was used as training data and testing data. NIST data is collected by the National Institute of Standards and Technology and contains about 210,000 compound 2D structures (format in .SDF) and their corresponding mass spectra (format in .msp). In addition to the NIST dataset, the SWGDRUG 3.11 dataset, comprising approximately 3,500 mass spectra of known illicit drugs, was also employed. The mass spectrum data of each compound were normalized, so that the sum of intensities of each mass spectrum is 100. The aim is to avoid the different scales of mass spectrums which output by different mass spectrometers will affect the training accuracy.

Ninety percent of the NIST datasets were randomly chosen as the training set for subsequent mass spectrometry and fingerprint prediction models. The remaining 10% was reserved as the testing set for evaluating the model's performance. Additionally, the SWGDRUG dataset was split into two equal halves, with one half designated for testing and the other half combined with the NIST training set to create a new training set.

Prior to testing actual sample mass spectra, the dataset was restructured since some test subjects were already present in the SWGDRUG database. These test subjects were excluded from the training set and the model was retrained.

In search of the similarity threshold, RDKit was used to extract the substructure of Cathinone. Compounds in the SWGDRUG containing this substructure were identified as Cathinone-type drugs, while the remaining compounds were considered non-cathinone-type drugs. One hundred instances were randomly selected for each category, and similarity calculations were performed with the enumerated databases separately.

Model training and hyperparameter setting

During NEIMS training, we developed our model following the guidelines provided on the NEIMS GitHub page, experimenting with various loss functions such as KL divergence, MSE, and cross-entropy. Ultimately, we opted for MSE as the most suitable loss function. The mask in hyperparameters was set to false. Regarding DeepEI training, we followed the default parameters.

Despite attempting early stop due to the extended training time, it proved ineffective, leading us to abandon its use.

Resource and Time Consumption

The enumeration of 420 million derivatives with 15 threads took approximately 15 hours. A total of 90 minutes to perform synthetic difficulty calculations by SCScore to filter functional groups with 15 threads. The speedup is linear to the number of threads used and can be easily scaled up. The training periods for NEIMS and DeepEI were 4.5 hours and 27 hours, respectively. Computing similarities and ranking for a tested analyte typically took around 1 minute.

Ranking

The similarity between the mass spectra of two molecules, A and B, is calculated using the cosine similarity calculation method, as employed in CFM-ID[6]. In cases where the mass spectrum of A contains N intensity values and that of B contains M intensity values, the two mass spectra are initially aligned through dynamic programming, resulting in the identification of Q pairs. k_a and k_b represent the m/z ratio values for molecules A and B, respectively, while p_a and p_b denote the intensities of compounds A and B at the respective m/z ratios, k_a and k_b. The calculation of cosine similarity between two mass spectra is represented by Equation 1.

$$cosine\ similarity_{MS}(a,b) = \frac{\left(\sum_{i=1}^{Q} {k_{ai}}^{0.5} p_{ai}^{0.5} \times {k_{bi}}^{0.5} p_{bi}^{0.5}\right)^2}{\sum_{n=1}^{N} \| k_{an} p_{an} \| \times \sum_{m=1}^{M} \| k_{bm} p_{bm} \|} \qquad (1)$$

In the calculation of mass spectrum similarity, in addition to cosine similarity, Jaccard similarity (equation 2) is also calculated. j_a and j_b are respectively represented as the set of m/z ratios of the top 30 intensities of compounds a and b in the mass spectrum.

$$Jaccard\ similarity_{MS}(a,b) = \frac{j_a \cap j_b}{j_a \cup j_b} \qquad (2)$$

The similarity calculation of the mass spectrum is the geometric mean of cosine similarity and Jaccard similarity (Equation 3).

$$score_{MS}(a,b) = \sqrt{cosine\ similarity_{MS}(a,b) * Jaccard\ similarity_{MS}(a,b)} \quad (3)$$

When comparing the similarity of fingerprints, Jaccard similarity (Equation 4) was used, where f_a and f_b represent the fingerprints of compounds a and b, respectively.

$$score_{FP}(a,b) = \frac{f_a \cap f_b}{f_a \cup f_b} \quad (4)$$

Finally, the weighted average score (Equation 5) was calculated by combining the mass spectrum score and the fingerprint score. This combined score, termed SMSF, represents the overall similarity between the analyte and the listed derivatives from the synthetic database.

$$score_{SMSF}(a,b) = 0.7 * score_{MS}(a,b) + 0.3 * score_{FP}(a,b) \quad (5)$$

The "ranking" for each compound in the testing set was determined by applying the mwf filters and assessing the similarity between each compound's real data and the predicted mass spectrum and fingerprint from the synthetic database model. The ranking indicates the similarity of a compound's real data to the predictions of the model in comparison to all the compounds in the synthetic database. The ranking proportion for the entire testing set was then determined. For instance, a ranking proportion of 0.9 for the NEIMS model ranking 100 indicates that 90% of the compounds in the testing set are ranked within the top 100 of the entire synthetic database based on the similarity between their real mass spectra and the one predicted by NEIMS. A higher

ranking proportion suggests a better similarity between the predicted results of the model and the real data, and hence, better performance of the model.

SMSF thresholding

From the SWGDRUG dataset, we randomly selected 100 cathinone-like drugs and 100 non-cathinone-like drugs. We then employed similarity calculations (specifically, SMSF) against the synthetic NPS database. For cathinone-like drugs, the resulting scores were used to establish a Gaussian distribution. In contrast, for non-cathinone-like drugs, we determined the highest similarity score found in the enumeration database to create a separate Gaussian distribution. The intersection point of these two Gaussian probability functions was defined as the threshold (see **Fig. S3**).

When assessing an unknown substance, this threshold was applied to evaluate the likelihood that the two substances are identical, even when the most similar compound in the database is identified.

Supplementary Figures

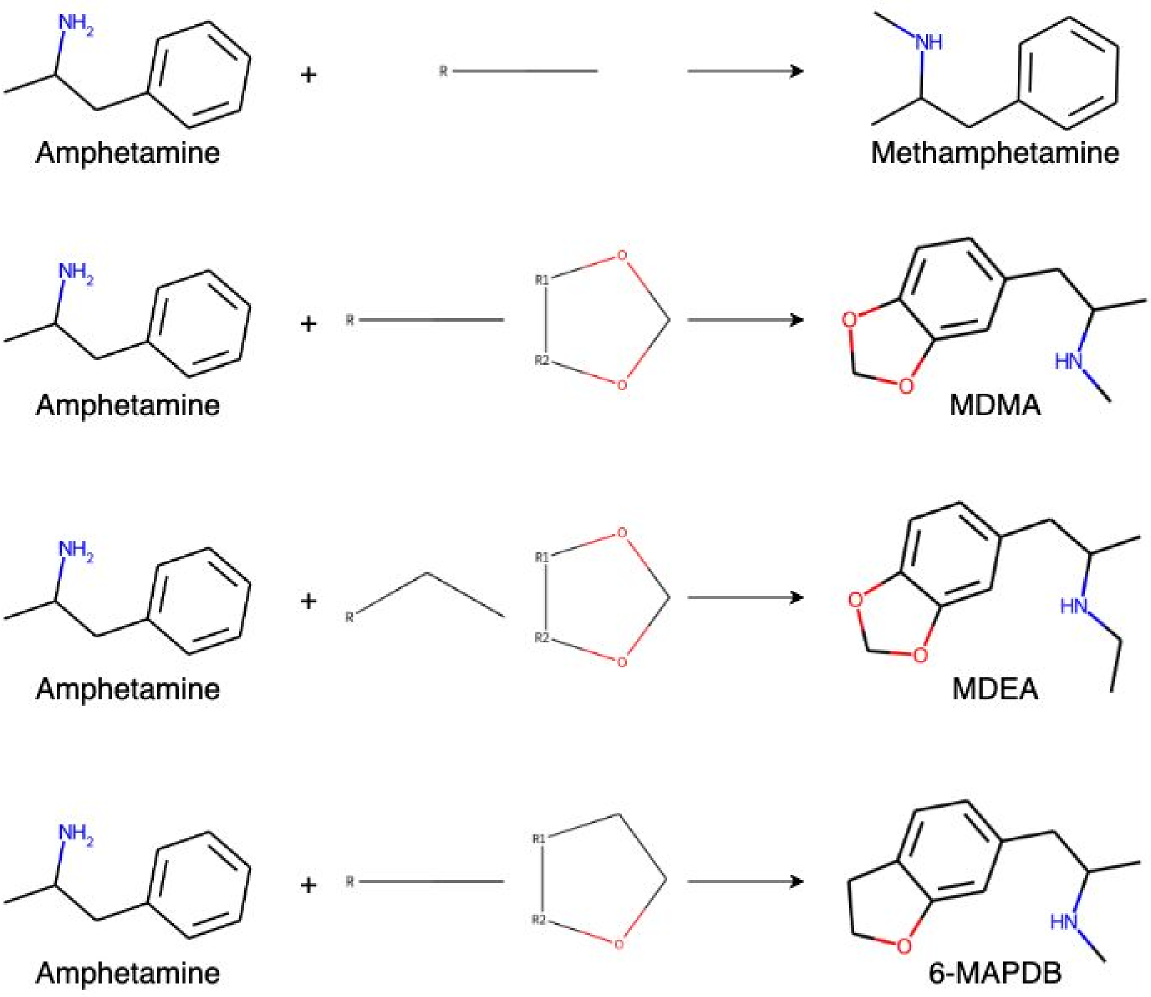

Figure S1. Methamphetamine, MDMA, MDEA, and 6-MAPDB belong to the amphetamine-type stimulant (ATS) class and can be produced by modifying the structure of Amphetamine.

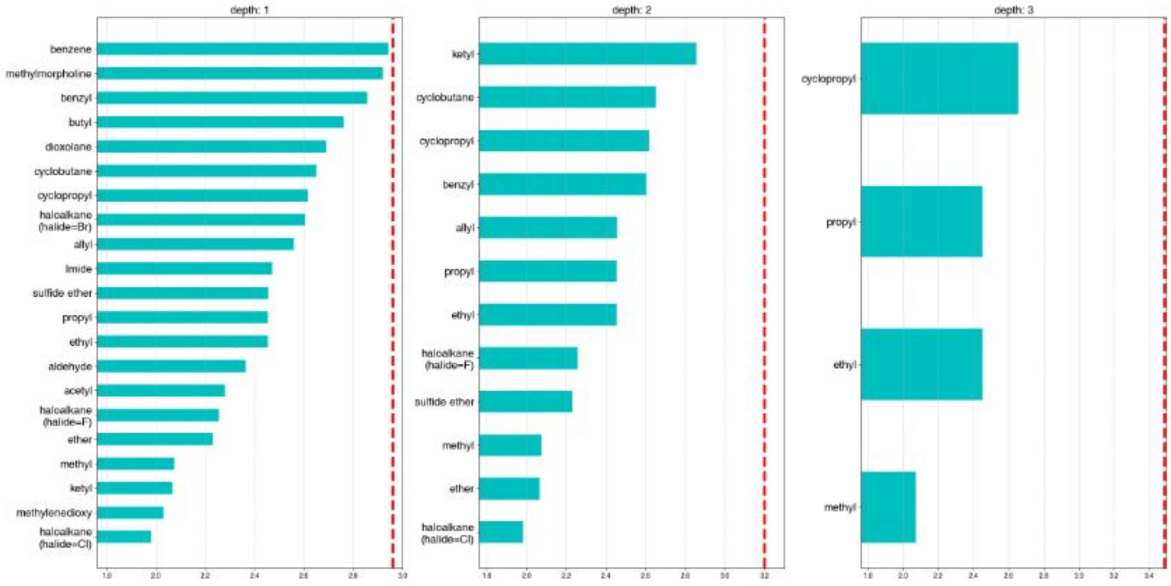

Figure S2. The functional groups in the figure are modifications added to the structure of cathinone based on the list of recognized cathinone-type drugs. The average synthesis difficulty of these functional groups is below the threshold for their respective depth. This ensures that existing cathinone-type illicit drugs won't be overlooked due to SCScore filtering.

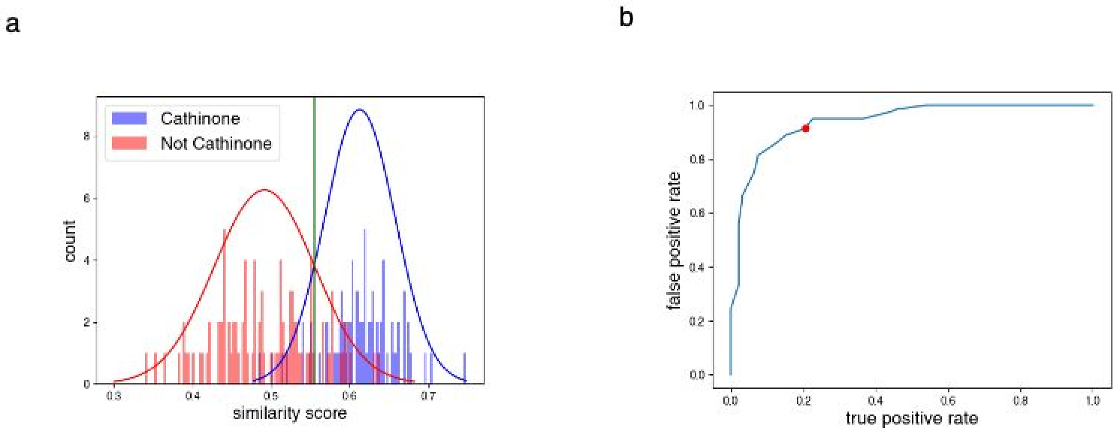

Figure S3. a Random selections were made from the SWGDRUG dataset, consisting of 100 Cathinone-type drugs and 100 other types of drugs. Subsequently, the matched similarity of Cathinone-type drugs and the top similarity score of other types of drugs were computed with our enumerated database. These score distributions were then mapped to Gaussian distributions, and the threshold was identified at the intersection of these two distributions, set at 0.55. **b** Utilizing diverse similarity scores as thresholds, true positive rates and false positive rates were calculated and presented as ROC curves. The red dot represents the threshold 0.55 obtained in a.

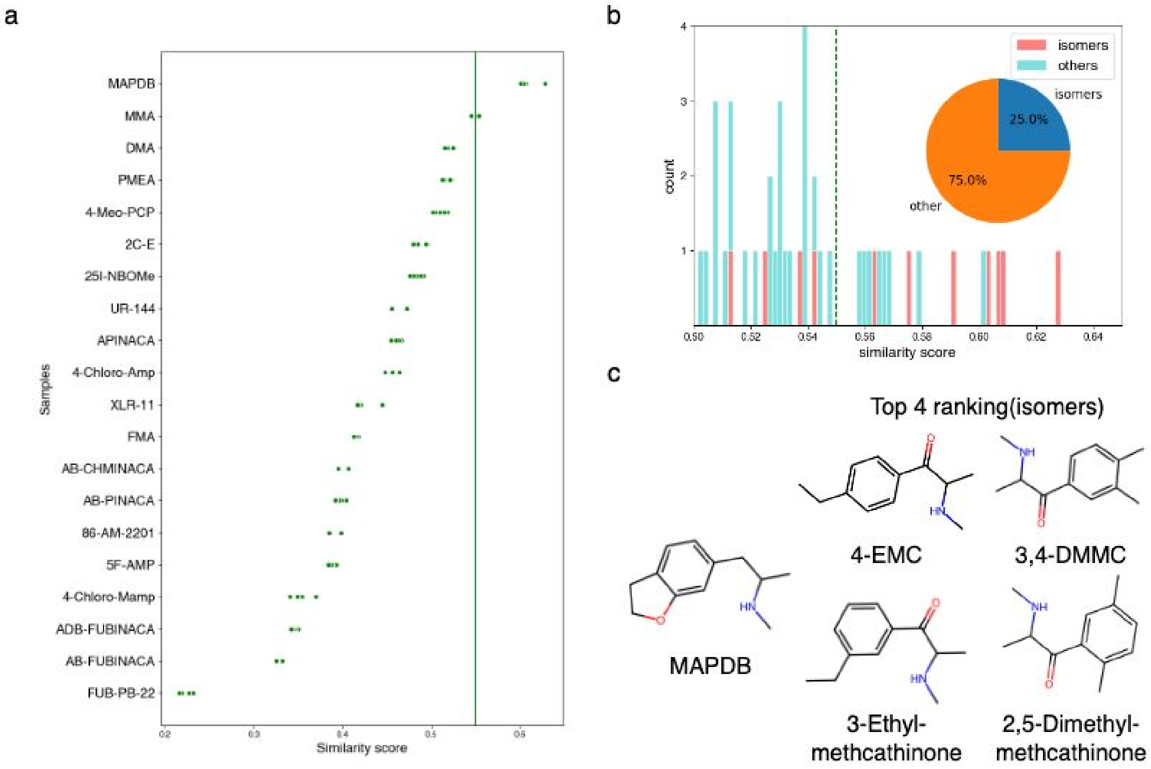

Figure S4. a The performance of real standard drugs not belonging to the cathinone-type illicit drugs was assessed in the cathinone enumeration database. The x-axis represents the similarity score, while the y-axis shows the names of the 20 illicit drugs. **b** Among the enumerated cathinones, the similarity score distributions for the not amphetamine-like drug MAPDB. The x-axis ranges from 0.5 to 0.65 in intervals of 0.0015. **c** The top four ranked compounds listed in the enumerated database are all isomers of MAPDB, as shown in their respective structures.

Supplementary Tables

Table S1. List of Functional Groups

	Number of substituents	Structure	Note
Acetal	4	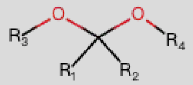	
Acetoxy	1	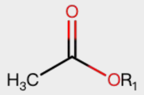	
Acetyl	1	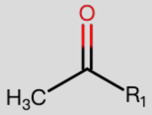	
Acetylide	1	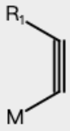	M stands for metal atom(Li, Na, K, Mg, Ca, De, Al)
Acid anhydride	2	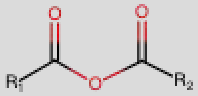	
Acryloyl	1	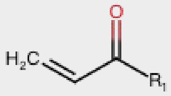	
Acyl azide	1	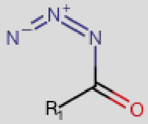	
Acyl halide	1	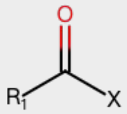	X represents halogen atom(Cl, F, Br, I)

Acylal	3	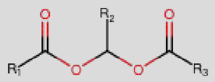	
Acylhydrazine	4	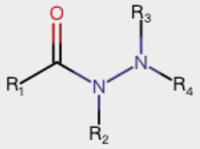	
Acyloin	2	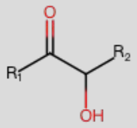	
Acylsilane	4	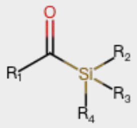	
Acylurea	1	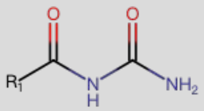	
Alcohol	1	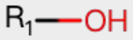	
Aldehyde	1	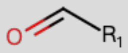	
Aldimine	2	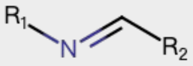	
Alkene	2	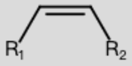	
Alkoxide	2	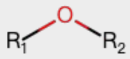	
Alkyl nitrites	1	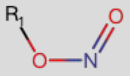	
Alkyne	2	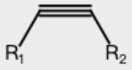	

Allyl	1	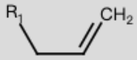	
Amide	1	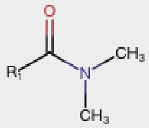	
Amidine	4	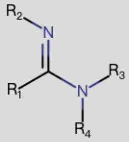	
Amidrazone	1	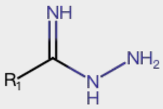	Hydrazide imides
Amidrazone	1	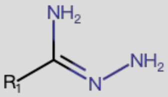	Amide Hydrazones
Aminal	6	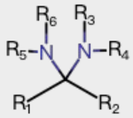	
Amine	3	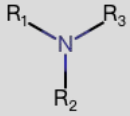	
Amine oxide	3	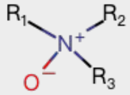	
Aminophosphine	6	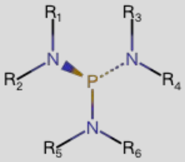	
Aminoxyl	3	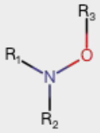	
Azide	1	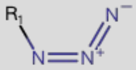	resonance structure 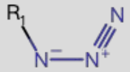

Azine	4	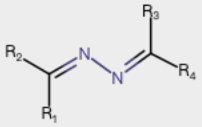	
Aziridine	5	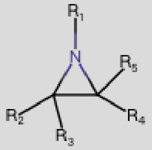	
Azo	2	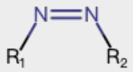	
Azole	4	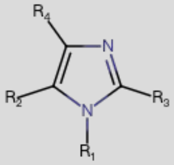	Imidazole
Azole	4	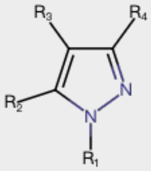	Pyrazole
Azole	3	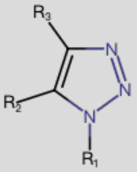	1,2,3-Triazole
Azole	3	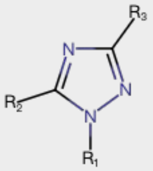	1,2,4-Triazole
Azole	2	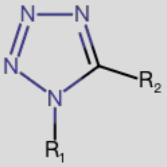	Tatrazole
Azole	3	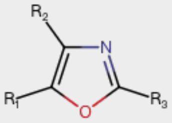	Oxazole
Azole	3	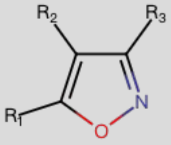	Isoazole

Azole	2	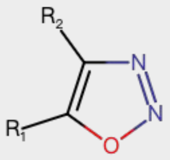	1,2,3-Oxadiazole
Azole	2	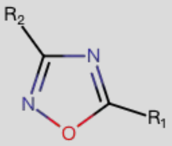	1,2,4-Oxadiazole
Azole	2	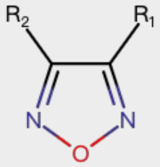	1,2,5-Oxadiazole
Azole	2	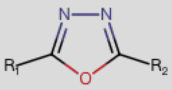	1,3,4-Oxadiazole
Azole	3	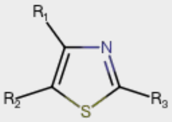	Thiazole
Azole	3	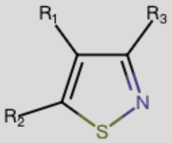	Isothiazole
Azole	2	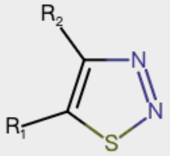	1,2,3-Thiadiazole
Azole	2	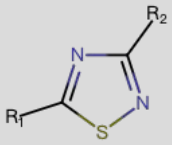	1,2,4-Thiadiazole
Azole	2	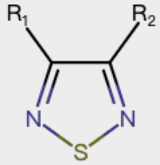	1,2,5-Thiadiazole

Azole	2	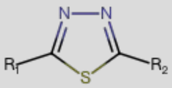	1,3,4-Thiadiazole
Azoxy	2	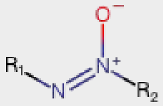	
Basic aluminium	1	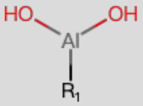	
Benzylidene acetal	2	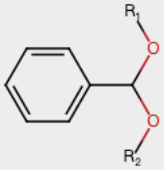	
Bisthiosemicarbazone	3	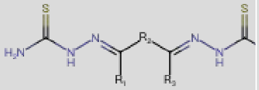	
Biuret	3	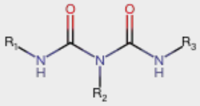	
Boronic acid	1	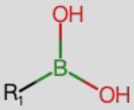	
Carbamate	3	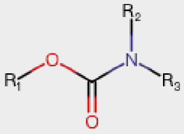	
Carbamoyl chloride	2	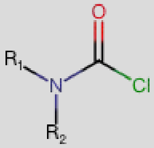	
Carbazide	2	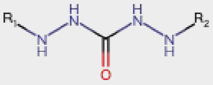	
Carbodiimide	2	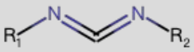	

Carbonate ester	2	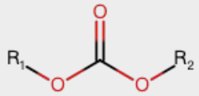	
Carbonyl	2	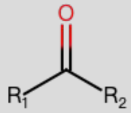	
Carboximidate	3	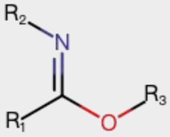	
Carboxylic acid	1	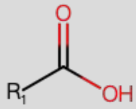	
Chloroformate	1	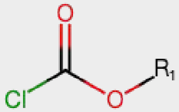	
Cyanate	1	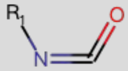	
Cyanate ester	1	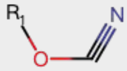	
Cyanimide	2	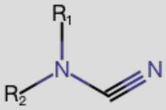	
Cyanohydrin	2	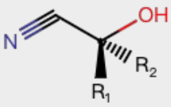	
Cyanomethyl	1	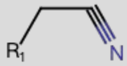	
Cyclopropyl	1	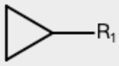	

Diazo	2	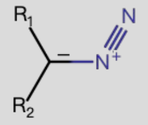	resonance structure: 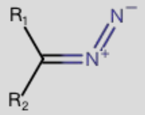
Dicarbonate	2	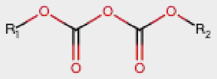	
Diketopiperazine	2	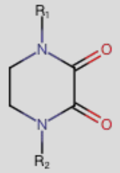	2,3-isomers
Diketopiperazine	2	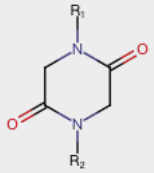	2,5-isomers
Diketopiperazine	2	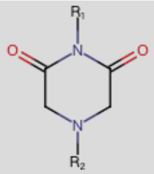	2,6-isomers
Dioxazolone	1	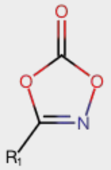	
Dioxirane	2	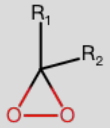	
Diphenyltriazene	3	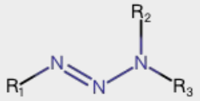	
Disulfide	2	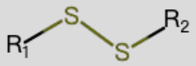	
Dithiocarbamate	3	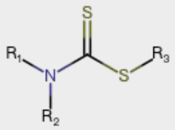	

Dithiol	2	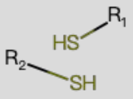	
Enamine	5	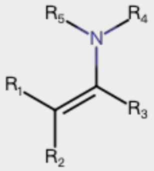	
Enediyne	4	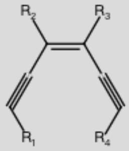	
Enol ether	4	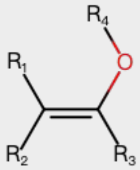	
Enone	4	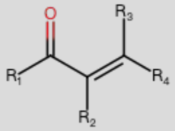	
Episulfide	4	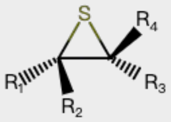	
Epoxide	4	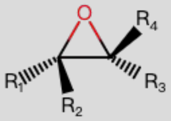	
Ester	2	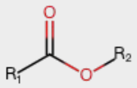	
Fluorosulfonate	1	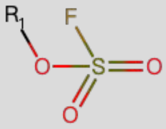	
Haloalkane	1	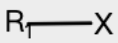	

Halohydrin	2	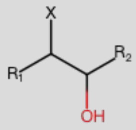	
Haloketone	3	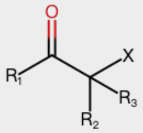	
Hemithioacetal	2	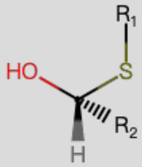	
Hydrazide	1	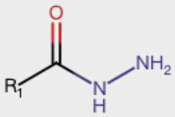	Carbohydrazide
Hydrazide	1	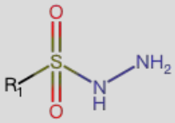	Sulfonohydrazide
Hydrazide	1	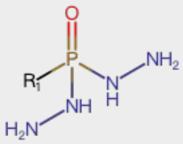	Phosphonic-dihydrazide
Hydrazone	2	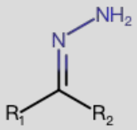	
Hydroperoxide	1	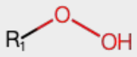	
Hydroxamic acid	2	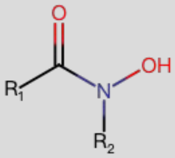	
Hydroxylamine	2	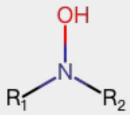	
Imide	3	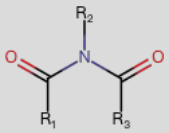	

Imidic acid	2	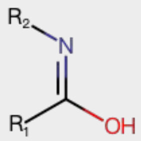	
Imidoyl chloride	2	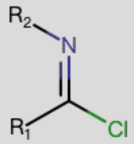	
Imine	3	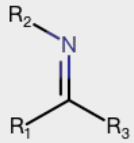	
Isocyanide	1	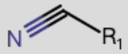	
Isodiazene	2	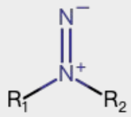	
Isodiazomethane	2	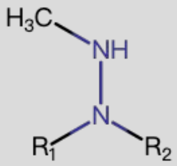	
Isothiocyanate	1	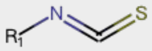	
Ketene	1	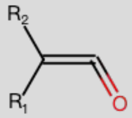	
Ketenimine	3	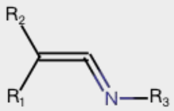	
Methanedithiol	2	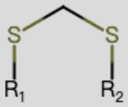	
Methine	2	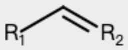	

Methylenedioxy	2	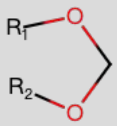	
Nitrate ester	1	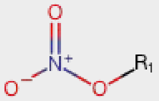	
Nitrile ylide	3	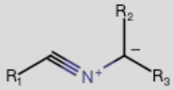	resonance structure: 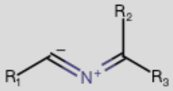
Nitrilimine	2	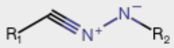	
Nitroamine	2	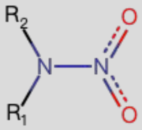	
Nitrolic acid	1	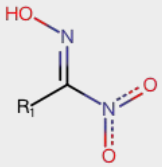	
Nitronate	2	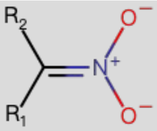	
Nitrone	3	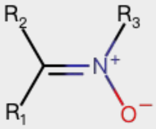	
Nitrosamine	2	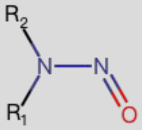	
Nitroso	1	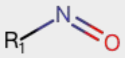	
Organic peroxide	2	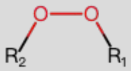	

Orthoester	4	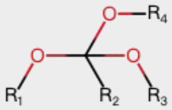	
Oxaziridine	3	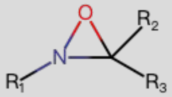	
Phosphaalkene	3	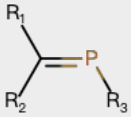	
Phosphaalkyne	1	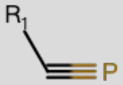	
Phosphate	3	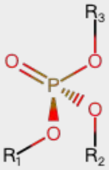	
Phosphinate	3	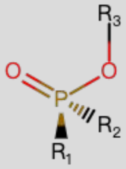	
Phosphine	3	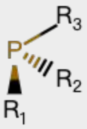	
Phosphine imide	4	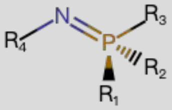	
Phosphine oxide	3	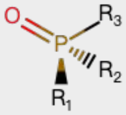	
Phosphinite	3	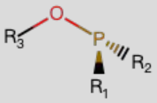	
Phosphinous anion	2	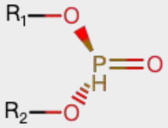	

Phosphite ester	3	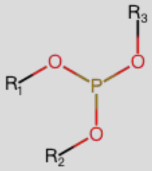	
Phosphonate	3	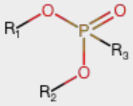	
Phosphonite	3	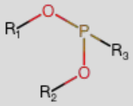	
Phosphonium	4	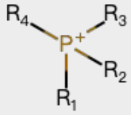	
Phosphoramidate	4	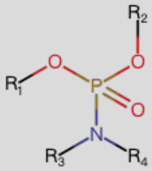	
Phosphoramide	6	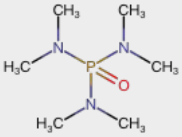	
Phosphoramidite	4	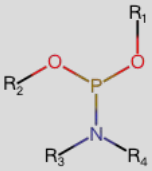	
Phosphorane	5	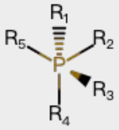	
Phosphorochloridate	2	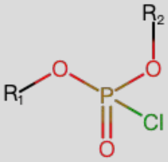	
Phosphochloridite	2	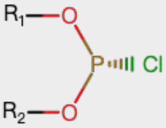	
Propenyl	1	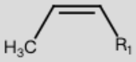	

Para-quinone methide	1	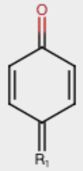	
Ortho-quinone methide	1	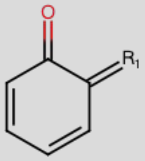	
Reductone	2	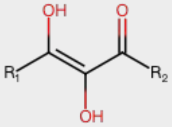	
S-Nitrosothiol	1	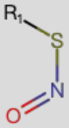	
Selenenic acid	1	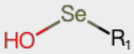	
Selone	1	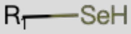	
Selenonic acid	1	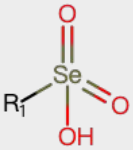	
Selone	2	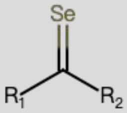	
Semicarbazide	5	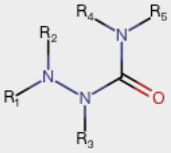	
Semicarbazone	5	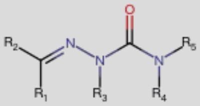	

Silyl enol ether	6	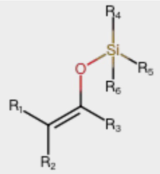	
Silyl ether	4	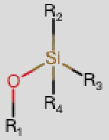	
Sulfamoyl fluoride	2	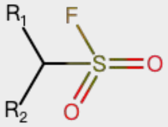	
Sulfenamide	3	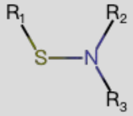	
Sulfenic acid	1	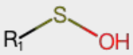	resonance structure: 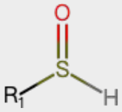
Sulfenyl chloride	1	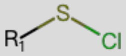	
Sulfide	1	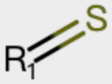	
Sulfilimine	3	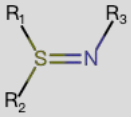	
Sulfinamide	3	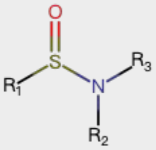	resonance structure: 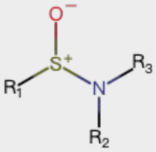
Sulfinic acid	1	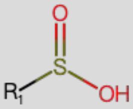	

Sulfite ester	2	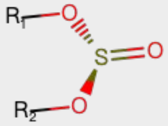	
Sulfonamide	3	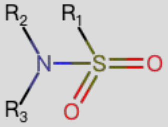	
Sulfonanilide	2	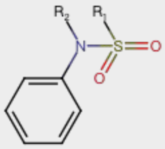	
Sulfonate	2	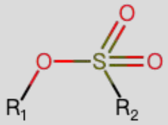	
Sulfone	2	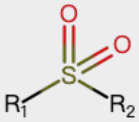	
Sulfonic acid	1	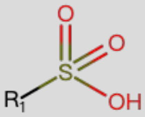	
Sulfonyl halide	1	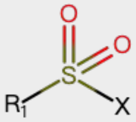	X represents halogen atom(Cl, F, Br, I)
Sulfoxide	2	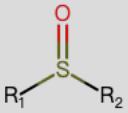	
Telluroketone	2	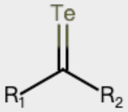	
Tellurol	1	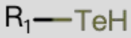	
Thial	1	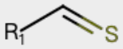	

Thioacetal	3	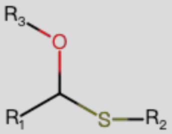	Monothioacetal
Thioacetal	3	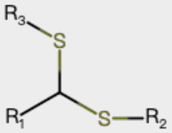	Dithioacetal
Thioacyl chloride	1	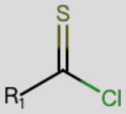	
Thioamide	3	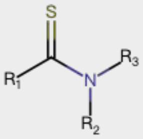	
Thiocarbamate	3	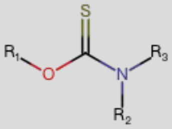	O-thiocarbamate
Thiocarbamate	3	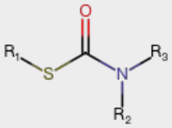	S-thiocarbamate
Thiocarboxylic acid	1	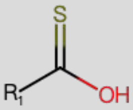	Carbothioic O-acid
Thiocarboxylic acid	1	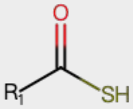	Carbothioic S-acid
Thioester	2	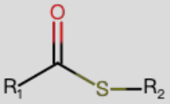	
Thioketal	4	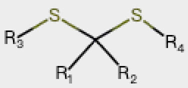	
Thioketene	2	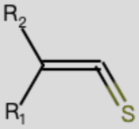	

Thioketone	2	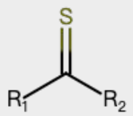	
Thiol	1	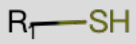	
Thiophosphate	3	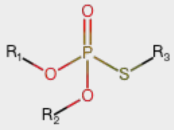	
Thiourea	4	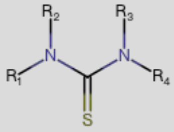	
Tosyl	1	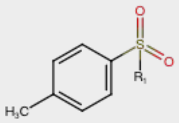	
Tosylate	1	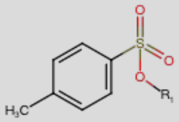	
Tosylhydrazone	2	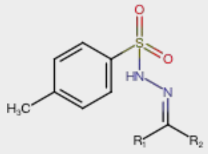	
Triazenes	3	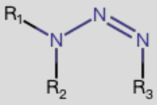	
Triuret	6	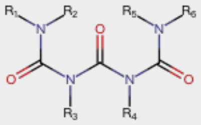	
Urea	4	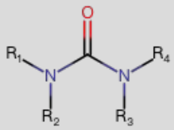	

Vinyl	1	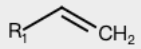	
Xanthate	1	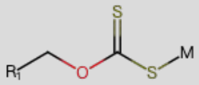	M = Na, K
Xanthate ester	2	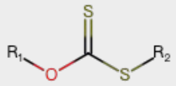	
Ynolate	1	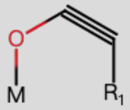	M stands for metal atom(Li, Na, K, Mg, Ca, De, Al)
Ynone	2	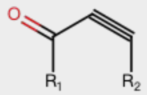	

Table S2. Table of Filter Rules[1-3]

Number	Name of the rules	Description
**1**	Bredt's rule	Bredt's rule states that a bridgehead carbon in a bridged or fused ring system cannot bear a double bond if it would result in a ring with fewer than eight atoms.
**2**	Element ratio filter	The molecules are first checked for the ratios N/C < 0.571, O/C < 0.666 and (N+O)/C < 1.0. Only those molecules passing the element ratio filter are kept.
**3**	Triple bonds in ring	The feature can significantly affect the chemical and physical properties of the compound, including its reactivity and stability.
**4**	Allene filter	Designed to avoid increased strain and reactivity due to the presence of two adjacent double bonds.
**5**	Acid Taut filter	Designed to avoid undergo rapid interconversion between different forms in solution, which may lead to potentially complicating analyses or reactions
**6**	Aminal filter	See [3] for detail
**7**	C=N filter	Designed to avoid compounds that may decompose at high temperatures.
**8**	Decarboxy filter	Implemented to exclude compounds prone to decarboxylation.
**9**	Enamine filter	See [3] for detail
**10**	Enol filter	Designed to avoid the increased reactivity or instability due to the presence of a highly reactive double bond adjacent to a hydroxyl group.
**11**	FC filter	See [3] for detail
**12**	Geminal filter	Designed to avoid the increased reactivity or instability due to steric hindrance or electronic effects.
**13**	Hemiacetal filter	Designed to avoid the increased reactivity or instability due to the presence of a potentially reactive hydroxyl group and an acetal-forming group.
**14**	Hemiaminal filter	Designed to avoid the increased reactivity or instability due to the presence of a potentially reactive hydroxyl group and an amine-forming group.

**15**	Het-Het filter	Designed to avoid the increased reactivity or instability under certain conditions due to the presence of electronegative atoms such as oxygen, nitrogen, sulfur, or halogens.
**16**	Het-Het-Het filter	Designed to avoid the increased reactivity or instability under certain conditions due to the presence of electronegative atoms such as oxygen, nitrogen, sulfur, or halogens.
**17**	Hetero-SR filter	See [3] for detail
**18**	IntraMol filter	Designed to avoid intramolecular interactions, such as strain or steric hindrance.
**19**	Mixed filter	Designed to avoid complex or unpredictable behavior due to interactions between the different groups.
**20**	Ortho filter	Designed to avoid the increased steric hindrance or electronic effects.
**21**	Polycyclic filter	Targets large polycyclic compounds that might decompose.
**22**	Topo I filter	Compounds interact with Topo I may interfere with its normal biological function, leading to potential adverse effects or alterations in cellular processes.
**23**	Non-aromatic nitro	Avoid the increased reactivity or instability due to the presence of the electron-withdrawing nitro group in a non-aromatic environment.
**24**	Non-aromatic halogen	Avoid the increased reactivity or instability due to the presence of the electronegative halogen atom in a non-aromatic environment.
**25**	Hetero-aromatic halogen	Avoid the increased reactivity or instability due to the presence of the electronegative halogen atom in a hetero-aromatic ring system.
**26**	Double bond in 4 ring	Avoid the geometric constraints, potentially leading to altered reactivity or undesired chemical behavior
**27**	Double bond in 3 ring	Avoid the geometric constraints, potentially leading to altered reactivity or undesired chemical behavior
**28**	Contain beta keto carboxyl	Avoid the presence of both a keto group and a carboxylic acid group in close proximity.
**29**	More than 7 SSSR rings	Aims to exclude compounds with many rings, which tend to be less volatile.

**30**	More than one SSSR ring of 8 or more atoms	Targets larger rings, which can lead to less volatile compounds.
**31**	Rings containing more than one bridge	Avoid theincreased strain and instability due to the limited flexibility of the bridged structure.
**32**	Unsaturations in bridges (except in bicyclooctene)	Avoid the increase the reactivity of the compound.
**33**	Ring systems which cannot be uniquely decomposed into rings and bridges	Avoid the complexity and potential for ambiguity in structural representation
**34**	Non-planar sp^2 systems (defined as systems X=C(Y)Z in which atom X is more than 0.15 Å outside the plane defined by atoms C, Y and Z)	The degree of planarity, which can influence the molecule's chemical behavior and interactions
**35**	Non-linear sp systems (systems A≡BC in which the angle ∠ABC<178.5°)	The non-linear arrangement of atoms in sp systems can result in steric hindrance. This hindrance can affect the compound's conformational flexibility and its ability to interact with other molecules.
**36**	Terminal sulfur atoms (except in thiourea and rhodanine)	Due to the highly reactive, participating in various chemical reactions such as oxidation, reduction, or nucleophilic substitution.

**37**	Single bonds between sulfur and oxygen	Targets bonds that might be reactive under the conditions of GC.
**38**	Single bonds between sulfur and nitrogen (except in aromatic sulfonamides)	Excludes such bonds, except in stable aromatic sulfonamides, where they could be less reactive.
**39**	More than 3 stereogenic carbons	Carbon atoms bond to four different substituents, resulting in chirality and the potential for multiple stereoisomers.
**40**	Multiple triple bonds separated by less than 8 bonds	Due to prone to decomposition or unwanted chemical reactions
**41**	No nitrogen or oxygen atoms	Nitrogen and oxygen atoms are frequently targeted sites for drug metabolism.
**42**	More than 5 halogens	Compounds with more than 5 halogens tend to be structurally complex and may have limited synthetic accessibility.
**43**	More than 2 aldehydes	Compounds with multiple aldehyde groups may undergo rapid metabolism, resulting in decreased systemic exposure and efficacy.
**44**	More than one methylidene group	Compounds with multiple methylidene groups tend to be structurally complex, which can present challenges in synthesis.
**45**	More than 2 unconjugated double bonds	The presence of multiple unconjugated double bonds could interfere with the compound's interaction with biological targets, reducing efficacy or causing off-target effects.
**46**	More than 3 basic amines	Compounds with multiple basic amine groups may exhibit poor solubility or stability in physiological conditions
**47**	XLogP>7.0	Targets compounds with a high LogP, as GC often uses non-polar stationary phases, and high LogP

		values might result in long retention times or adsorption issues.
**48**	More than 10 hydrogen bond acceptors	Compounds with multiple hydrogen bond acceptors may undergo rapid metabolism, leading to decreased systemic exposure and reduced efficacy.
**49**	More than 5 hydrogen bond donors	Excessive hydrogen bond donors and acceptors can interfere with the compound's ability to bind to specific receptors or enzymes.
**50**	More than 11 rotatable bonds	Aims to exclude compounds with many rotatable bonds, as they often exhibit lower volatility.
**51**	Enol ethers, acyl-halides, anhydrides, beta-heterosubstituted carbonyls, perhalo-ketones, unsubstituted hexane chains, or halopyrimidines	Designed to filter out compounds with potentially reactive functional groups.
**52**	A heteroatom adjacent to (but not part of) an sp system	The presence of a heteroatom adjacent to an sp system can introduce steric hindrance, affecting the spatial arrangement of the molecule. This hindrance may interfere with the compound's ability to bind to biological targets.
**53**	Cyclic sulfur bonded to fused or bridgehead nitrogens	Compounds containing cyclic sulfur bonded to fused or bridgehead nitrogens tend to be structurally complex.
**54**	Heteroaromatic halogens	The presence of multiple halogens can interfere with the compound's interaction with biological targets.
**55**	Non-aromatic halogens (except in aromatic trihalomethyl groups)	Halogens may exhibit increased reactivity or disrupt essential biological processes

**56**	Double bonds to terminal atoms (except in sulfones)	Double bonds to terminal atoms can exhibit increased reactivity, making them susceptible to oxidation or other chemical transformations.
**57**	Chains of 3 or 4 aromatic nitrogens (except in triazoles and tetrazoles)	Compounds containing chains of aromatic nitrogens tend to be structurally complex.

Reference

(1) Virshup, A. M.; Contreras-García, J.; Wipf, P.; Yang, W.; Beratan, D. N. Stochastic Voyages into Uncharted Chemical Space Produce a Representative Library of All Possible Drug-Like Compounds. *Journal of the American Chemical Society* **2013**, *135* (19), 7296-7303. DOI: 10.1021/ja401184g.
(2) Blum, L. C.; Reymond, J.-L. 970 million druglike small molecules for virtual screening in the chemical universe database GDB-13. *Journal of the American Chemical Society* **2009**, *131* (25), 8732-8733.
(3) Fink, T.; Reymond, J.-L. Virtual exploration of the chemical universe up to 11 atoms of C, N, O, F: assembly of 26.4 million structures (110.9 million stereoisomers) and analysis for new ring systems, stereochemistry, physicochemical properties, compound classes, and drug discovery. *Journal of chemical information and modeling* **2007**, *47* (2), 342-353.
(4) Heid, E.; Greenman, K. P.; Chung, Y.; Li, S.-C.; Graff, D. E.; Vermeire, F. H.; Wu, H.; Green, W. H.; McGill, C. J. Chemprop: A Machine Learning Package for Chemical Property Prediction. *Journal of Chemical Information and Modeling* **2024**, *64* (1), 9-17. DOI: 10.1021/acs.jcim.3c01250.
(5) Landrum, G. RDKit: Open-source cheminformatics. 2006. *Google Scholar* **2006**.
(6) Djoumbou-Feunang, Y.; Pon, A.; Karu, N.; Zheng, J.; Li, C.; Arndt, D.; Gautam, M.; Allen, F.; Wishart, D. S. CFM-ID 3.0: significantly improved ESI-MS/MS prediction and compound identification. *Metabolites* **2019**, *9* (4), 72.
